# Reef larval recruitment in response to seascape dynamics in the SW Atlantic

**DOI:** 10.1038/s41598-022-11809-1

**Published:** 2022-05-11

**Authors:** Ana Carolina de Azevedo Mazzuco, Angelo Fraga Bernardino

**Affiliations:** Benthic Ecology Group, Department of Oceanography and Ecology, Universidade Federal do Espírito Santo, Av. Fernando Ferrari, 514, Vitória, ES 29075-910 Brazil

**Keywords:** Ocean sciences, Biodiversity, Community ecology, Ecological modelling

## Abstract

Advances in satellite observation have improved our capacity to track changes in the ocean with numerous ecological and conservation applications, which are yet under-explored for coastal ecology. In this study, we assessed the spatio-temporal dynamics in invertebrate larval recruitment and the Seascape Pelagic Habitat Classification, a satellite remote-sensing product developed by the Marine Biodiversity Observation Network (MBON) and delivered by the US National Oceanic and Atmospheric Administration to monitor biodiversity globally. Our ultimate goal was to identify and predict changes in coastal benthic assemblages at tropical reefs in the SW Atlantic based on integrated pelagic conditions, testing the use of MBON Seascape categorization. Our results revealed that the pelagic Seascapes correlated with monthly and seasonal variations in recruitment rates and assemblage composition. Recruitment was strongly influenced by subtropical Seascapes and was reduced by the presence of warm waters with high-nutrient contents and phytoplankton blooms, which are likely to affect reef communities in the long term. Recruitment modeling indicates that Seascapes may be more efficient than sea surface temperature in predicting benthic larval dynamics. Based on historical Seascape patterns, we identified seven events that may have impacted benthic recruitment in this region during the last decades. These findings provide new insights into the application of novel satellite remote-sensing Seascape categorizations in benthic ecology and evidence how reef larval supply in the SW Atlantic could be impacted by recent and future ocean changes.

## Introduction

Seascapes have experienced unprecedented changes during the last decades, with records of altered conditions from the surface to the deep areas of the ocean^1–4^. As seascape modification intensifies and repeats itself at different regions of the ocean, scientists will need to anticipate biodiversity patterns and understand how ecosystem processes and species populations will behave across these distinct marine conditions^5,6^. Although it involves a lot of monitoring effort, seascape changes can be tracked by mapping variations in different components of marine ecosystems (e.g., benthic and pelagic interactions^7^ or essential ocean variables^8^) and by identifying their levels of association in the long-term^9^. Seascape ecology is an oriented framework with many tools to help marine spatial planning and conservation efficiency at large scales^10,11^. This approach could improve our capacity to predict biodiversity loss and recovery, and thus, provide valuable information to maximize management and mitigate global ocean change with more specificity^12,13^.

Under a changing ocean, the reduction of local populations is the first step towards species extirpation and an alarm to potential marine biodiversity loss at larger scales^14,15^. Although vulnerable populations are commonly identified through adult mortality, reduction of offspring supply can also be used as an alert for potential species disappearance and change in the health status of populations and communities (e.g.^16,17^). Many marine organisms have a life cycle with an intermediate larval stage and local biodiversity in these marine ecosystems is strongly linked to oceanographic and ecological processes that influence the larval supply and recruitment (e.g.,^18,19^). Processes influencing benthic recruitment may act at distinct spatial and temporal scales related to pelagic conditions that affect planktonic larval development and supply rates^20–22^. Thus, lower or higher numbers of benthic recruits over an area may be a response to changes in pelagic seascapes, which will have later consequences on the overall biodiversity of that region^23,24^. For these benthic species, recruitment also requires surviving strong local post-settlement pressures, such as competition, predation, and unfavorable environmental conditions that impact juvenile abundances^25,26^.

Monitoring larval dynamics can potentially anticipate ecological responses to changes in marine seascapes, but it is a challenging task and often neglected by long-term observing programs. For rocky shore and coral reef populations, which are widely studied marine ecosystems with respect to larval dynamics, variations in larval recruitment can be estimated using pelagic variables such as sea surface temperature, chlorophyll-a concentrations, current velocities, wave height, and the occurrence of oceanographic features such as upwellings, cold fronts, and storms^27–31^. Several studies indicate that the early life stages of benthic invertebrates are highly vulnerable to a changing ocean, where even small variations in pelagic primary productivity or temperature fluctuations, for instance, may have strong impacts on benthic recruitment (e.g.^32^). Modeling larval dynamics from pelagic variables could be a useful approach to develop ecological metrics of benthic community change and, in turn, applied to monitor the coastal ecosystems.

Currently, many oceanographic variables are readily available online in a wide range of spatial and temporal resolutions that are derived from in situ monitoring networks or satellite products^33,34^. These ocean observing databases are rich, diverse, and can be easily retrieved to develop panoramas of the pelagic systems at multiple levels and for different purposes. Among them, the Seascape Pelagic Habitat Classification developed by the Marine Biodiversity Observation Network in partnership with the US Integrated Ocean Observation System (IOOS) and the National Oceanic and Atmospheric Administration (NOAA) is a novel satellite remote sensing approach that translates spatial and temporal variations in seascapes from multiple changes in pelagic conditions (CoastWatch/OceanWatch)^35,36^. The MBON Seascape classification is obtained from dynamic fields of sea surface satellite measurements and modeled data that integrates a set of ocean variables (sea surface temperature, sea surface salinity, absolute dynamic topography, chromophoric dissolved organic material, surface chlorophyll-a, and normalized fluorescent line height) into a categorization system of 33 water masses. This categorization is designed to support biogeographical assessments and marine observation networks, providing an environmental tool to track changes in marine habitats with relatively high resolution. By integrating key pelagic conditions and states, the MBON Seascape classification goes beyond single-variable categorizations (sea surface temperature anomaly; e.g.,^37^) by reflecting the quality and extent of different oceanographic domains or features. This global ocean classification is a promising tool to assess and predict pelagic-driven biological processes (phytoplankton community composition, e.g.,^38^).

Considering that larval dynamics is key to the ecology of benthic communities and strongly dependent on oceanographic conditions, we examined whether changes in MBON integrated Seascape Pelagic Habitat Classification (hereafter Seascapes) can be associated with larval recruitment at tropical intertidal reefs and could be applied to forecast recruitment variability. The study was carried out at a Brazilian LTER site (HCES) on the coastal SW Atlantic in a marine protected area located in the Eastern Brazil Marine Ecoregion. The coastal reefs in this area are covered by dense and rich macroalgal beds, whose range expands from the intertidal to mesophotic depths, hosting high abundances of invertebrate recruits^39–41^. Like many other sites along the tropical Brazilian coast, this region has experienced significant warming trends during the last four decades (1970–2010;^42^) and previous work indicated that larval recruitment and adult intertidal reef assemblages in this region are vulnerable to these periods of high sea temperatures^39,40^. These coastal changes to warmer seascapes may lead to negative effects on reef biodiversity and should be mapped in order to improve ecological forecast and conservation actions in the region. There has been a significant advance in long-term monitoring of the coastal ecosystems in the SW Atlantic in the last decades, particularly in the tropical reefs^43,44^. However, the scarcity of basic knowledge about reef dynamics in particular regions and punctual ecological modeling efforts imposes several challenges to reef management, such as preventing the anticipation of potentially harmful conditions and forecasting ecosystem changes.

Using the LTER HCES dataset that represents regional recruitment trends^39,40^, we quantified recruitment across multiple temporal scales (years, seasons, and months) for several reef invertebrate species that use macroalgal beds as settlement substrate. Recruitment was sampled in the Sargassum beds, which are the dominant macroalgae at the intertidal fringe and tidal pools at these reefs^40^. We then tested the association between recruitment patterns (abundance and composition of species) and Seascapes, identifying the Seascape classes that were key to recruitment variability. We then evaluated the effectiveness of Seascapes in predicting recruitment success (i.e. higher or lower abundance and diversity), when compared to a single-variable approach using sea surface temperature as a driver of larval dynamics in this region^39^. We also tested if past Seascape dynamics could indicate periods of decreased recruitment potential by revisiting the previous 16 years (2003 to 2019) of monthly Seascape conditions in the area of study. Our hypothesis was that recruitment rates (i.e. abundance) and taxonomic composition would be highly correlated to changes in Seascapes through time, reflecting both local and regional variability in pelagic habitats. This study provides new insights into the application of pelagic Seascape classifications to the supply-side ecology at macroalgal tropical reefs in the tropical South Atlantic and reveals how these benthic communities may be highly vulnerable to a warmer ocean. We believe that identifying environmental drivers for marine assemblages in these reef ecosystems is the first step towards improving ecological predictions and could be important subside for planning and mitigation of environmental impacts at SW Atlantic reefs and coastal MPAs^45–47^.

## Results

### Seascapes

**Figure 1:**
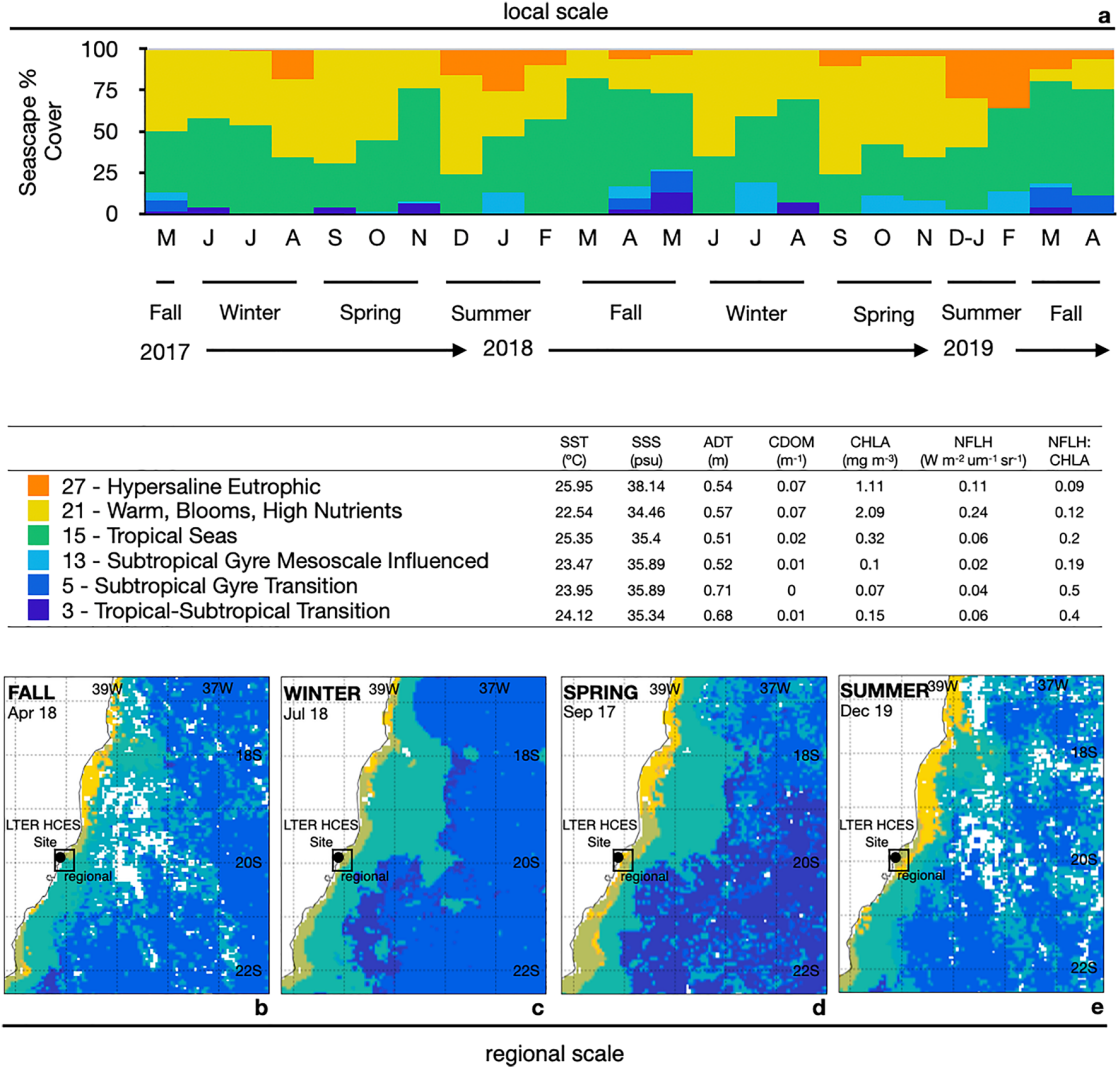
Monthly (**a**) and seasonal (**b**–**e**) variations in the Seascapes % Cover (local scale ~ 30 km coastline, 465 km^2^; and regional scale ~ 700 km coastline, 357,500 km^2^) between May 2017 to April 2019. Mean values of oceanographic variables that identify Seascape water masses (classes). *SST* sea surface temperature, *SSS* sea surface salinity, *ADT* absolute dynamic topography, *CDOM* chromophoric dissolved organic material, *CHLA* chlorophyll-a, *NFLH* normalized fluorescent line height.

According to the MBON pelagic Seascape categorization, six Seascape classes were detected within the study region at both local scale (~ 30 km coastline, 465 km^2^) and regional scale (~ 700 km coastline, 357,500 km^2^), including the Tropical-Subtropical Transition (class 3), Subtropical Gyre Transition (class 5), Subtropical Gyre Mesoscale Influenced (class 13), Tropical Seas (class 15), Warm, Blooms, High Nutrients (class 21), and Hypersaline Eutrophic (class 27) (Fig. 1). These different pelagic seascapes classes occur at distinct areas of the coast, varying depending on the distance from the shoreline and latitude (Fig. 1). Temporal variations in Seascape classes at local and regional scales were correlated (correlation analysis results, df = 22, t = 3.04, p = 0.0059, R = 0,54; see Supplementary Fig. S2). Overall, the Seascape categories in this region are characterized by high sea surface temperatures (SST > 22.5 °C), high sea surface salinity (SSS > 34), and calm waters (absolute dynamic topography ADT 0.52 to 0.71). We found a strong variation in the dissolved organic matter and chlorophyll-a concentrations (chromophoric dissolved organic material CDOM 0 to 0.07, surface chlorophyll-a CHLA 0.07 to 2.09) and fluorescence (normalized fluorescent line height NFLH 0.02 to 0.24) across the Seascape classes (Fig. 1). Lowest CDOM and CHLA values were observed during the predominance of subtropical gyre seascapes (classes 5 and 13, respectively), whereas eutrophic and warm, blooms, high nutrient seascapes had the highest nutrients and chlorophyll (classes 21 and 27, respectively; Fig. 1). Oligotrophic Seascapes (classes 13, 5, and 3) occupy a larger area of the coast at regional scales and occur more often at offshore sites (Fig. 1).

The Seascapes Warm, Blooms, High Nutrients, and Tropical conditions were dominant with over 70% of coverage during the study period (Fig. 1). As expected, the predominance of Seascape classes differed seasonally and monthly (seasons F = 7.64, p = 0.01, month F = 1.82, p = 0.04, Table 1; p < 0.05, See Supplementary Table S3). Subtropical conditions (Seascape classes 3, 5, and 13) were more frequent during the fall (Mar-May) when the pelagic conditions were similar and more influenced by southern Atlantic water masses (Fig. 1). On the other hand, Hypersaline Eutrophic conditions occurred frequently during the summer (Dec-Fev) at both local and regional spatial scales, with the predominant influence of tropical warm currents (Fig. 1). The seasonal variability on Seascape conditions did not change at the annual scale (F = 0.62, p = 0.57, Table 1).

**Table 1:** Results of the 3-way permutational multivariate analysis of variance (PERMANOVA) to compare the differences in Seascape % Cover (number of pixels) per class between months, seasons.

Seascapes					
**2017–2019**	*df*	**SS**	**MS**	**F**	*p*
Year	1	0.01	0.01	0.62	0.57
Season	3	0.64	0.21	7.64	**0.01**
Month	8	0.41	0.05	1.82	**0.04**
Year*Season	3	0.21	0.07	2.51	0.08
Residuals	8	0.22	0.02		
Total	23	1.51			

**2003–2019**	*df*	**SS**	**MS**	**F**	*p*
Year	1	0.28	0.28	5.59	**0.01**
Season	3	2.45	1.15	22.76	**0.01**
Month	8	1.25	0.15	3.10	**0.01**
Year*Season	3	0.30	0.10	2.01	**0.04**
Residuals	176	8.89	0.05		
Total	191	14.19			

Analysis included the sampling years (2017–2019) and the previous 16 years (2003–2018). F for statistic, significant results (p < 0.05) are in bold.

### Trends in recruitment

**Figure 2:**
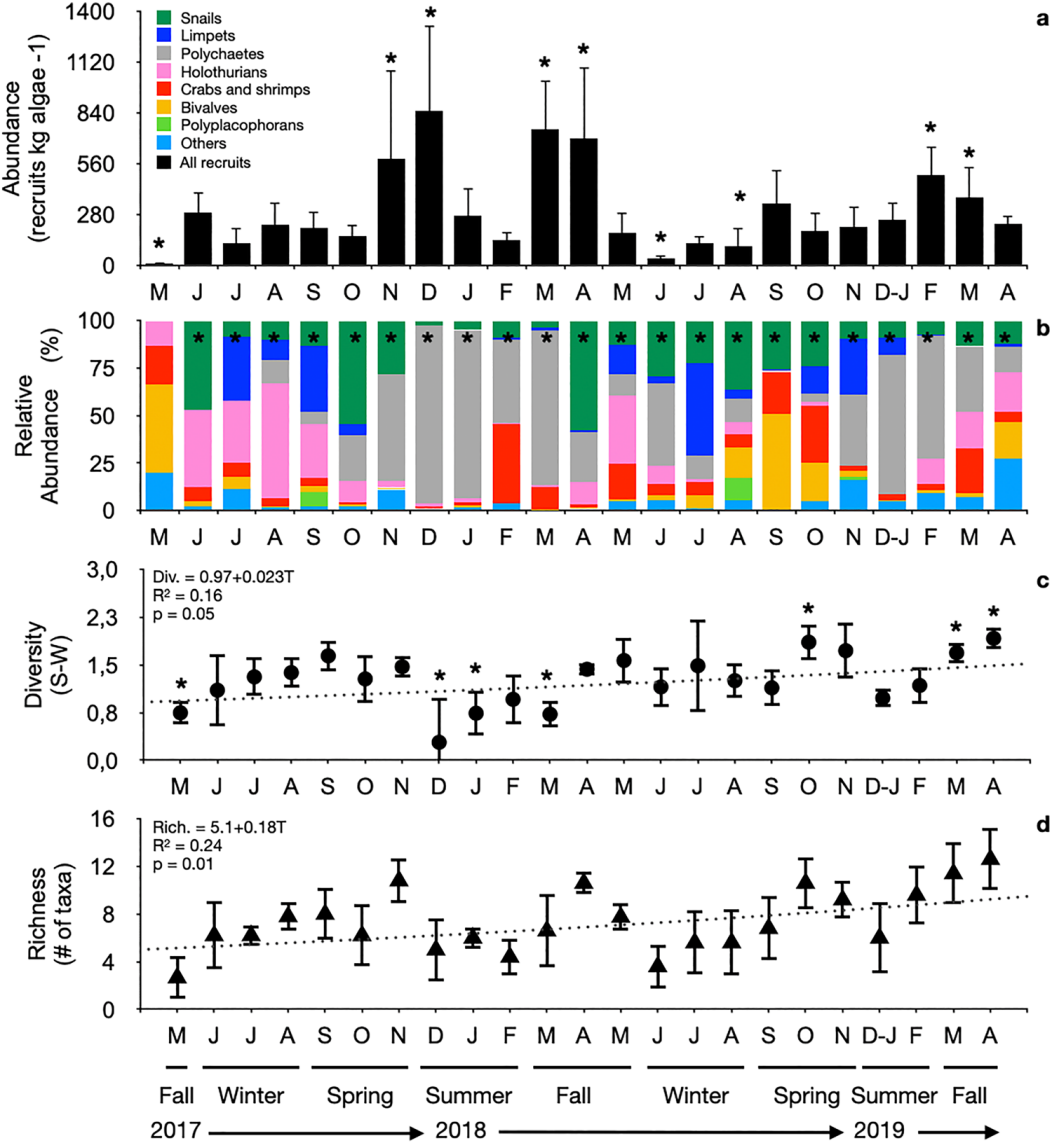
Monthly recruitment abundance (total abundance of recruits; (**a**), assemblage composition (number of recruits per taxa; (**b**), diversity (Shannon–Wiener Index S-W; (**c**), and richness (number of species/taxa; (**d**) (per kg of algae) at the study site. Note: averages and standard deviations are represented by columns/symbols and error bars respectively; color bars represent relative frequency per taxa (%); the dotted lines represent the linear trends in recruitment from the regression analysis; and significant monthly differences according to ANOVAs and PERMANOVAs are highlighted (*).

Over 60 taxa recruited on the Sargassum beds during the 24-month period (See Supplementary Table S4), with a predominance of polychaete (32%), gastropods (30%), and holothurians (14%) (Fig. 2; see Supplementary Table S4). Recruitment was continuous over the year with an average of 2 to15 taxa recruiting simultaneously every month (Fig. 2). Recruit abundance, composition, diversity, and richness varied significantly between seasonal and monthly time scales (p < 0.01, Table 2). We observed a lower abundance of recruits during winter (May or Jun), lower diversity during summer (Dec-Jan), and higher richness during fall and spring (Tukey HSD p < 0.05, see Supplementary Table S5). Recruits peaked in abundance on two observed events that occurred during summer 2017 (Nov-Dec) and fall 2018 (May-Jun) when abundance was almost twice above average (Tukey HSD p < 0.005, see Supplementary Table S6; Fig. 2). The lowest diversity of recruits was registered during the peak abundance in summer 2017 (Dec) and spring (Mar) (Tukey HSD p < 0.001, see Supplementary Table S5 and S6; Fig. 2). The diversity and richness of recruits increased from the years 2017 to 2019 (Fig. 2) with significant between-year variability (diversity F = 23.95, p < 0.0001, richness F = 7.19, p = 0.0084, Table 2).

**Table 2:** Results of 3-way analyses of variance and permutational analysis of variance to compare the differences in recruit assemblage abundance (total number of recruits), composition (number of recruits per taxa), diversity (Shannon–Wiener Index S-W), and richness (number of species/taxa) between months, seasons, and years.

	***df***	**SS**	**MS**	**F**	***p***
**Abundance**					
Year	1	1.24	1.24	1.41	0.2373
Season	3	14.50	4.83	5.47	**0.0011**
Month	2	10.15	5.07	6.03	**0.0033**
Year*Season	3	7.00	2.3	2.77	**0.0451**
Residuals	105	88.36	0.84		
**Assemblage composition**					
Year	1	0.16	0.16	10.47	**0.01**
Season	3	0.34	0.11	7.21	**0.01**
Month	2	0.03	0.03	2.51	**0.01**
Year*Season	3	0.22	0.07	4.78	**0.01**
Residuals	103	1.63	0.01		
Total	111	2.40			
**Diversity**					
Month	1	3.72	3.72	23.95	** < 0.0001**
Season	3	6.22	2.07	13.33	** < 0.0001**
Month	2	1.81	0.91	5.85	**0.0039**
Year*Season	3	2.37	0.79	5.09	**0.0024**
Residuals	105	16.33	0.15		
**Richness**					
Site	1	52.99	52.99	7.19	**0.0084**
Season	3	189.31	63.10	8.57	** < 0.0001**
Month	2	29.33	14.67	1.99	0.1414
Year*Season	3	135.91	45.30	6.15	** < 0.0001**
Residuals	105	772.86	7.36		

F for statistic, significant results (*p* < 0.05) are in bold.

We observed an annual variability in the recruit assemblage composition (F = 10.47, p = 0.01, Table 2 and S6; Fig. 2), with a lower number of holothurians in the second year of sampling (F = 15.17, p = 0.0001, see Supplementary Table S8 and S9; Fig. 2 and Supplementary Fig. S3). Seasonality was an important component of recruit taxonomic composition (p < 0.05, Table 2 and Supplementary Table S6; Fig. 2), but temporal variability was taxa specific (Fig. 2 and Supplementary Fig. S3). Monthly variations were significant for snails and limpets, and polychaetes had a higher number of recruits during summer and fall (Nov–Apr) (p < 0.05, see Supplementary Table S8 and S9; Fig. 2 and S3). Crabs and shrimps had more recruitment during the fall (Mar), whereas limpets, holothurians, and bivalves did not show consistent seasonal recruitment during the time sampled (p < 0.05, see Supplementary Table S8 and S9; Fig. 2 and Supplementary Fig. S3). Despite monthly and year differences in the number of recruits sampled, the number of samples represented well the local species diversity after the first months of study (see Supplementary Fig. S4).

### Association between recruitment and Seascapes

The abundance and richness of benthic recruits were temporally correlated with Seascapes (Tables 3 and Supplementary Table S10). Peaks in recruit abundances occurred one month after intrusions of Subtropical Gyre Transitions Seascape conditions (r = 0.51, p < 0.01, Table 3), and when Tropical waters were frequent in the region (r = 0.49, p < 0.01, Table 3). The peaks in abundance described above were particularly associated with a higher dominance of snails (r = 0.64, r = 0.41, p < 0.01, Table 3). Other taxa peaked following the dominance of Hypersaline Eutrophic waters, including crab and shrimp recruits (r = 0.44, p < 0.01, Table 3). Peaks in bivalve recruitment occurred after the onset of Subtropical Gyre Mesoscale Influence (r = − 0.45, p < 0.01, Table 3). Therefore, recruit richness increased when coastal waters were more heterogeneous and with a weakening of the Warm, Blooms, High Nutrients Seascape (r = − 0.45, p < 0.01, Table 3).

**Table 3:** Significant results of the cross-correlation analysis (CCA) comparing recruitment and Seascape variability (% Cover) and correspondent generalized additive models (GAM).

	CCA		GAM				
	r	lag	t	*p*	F	*p*	R-sq
**Abundance**							
Total * Seascape 5	0.51	− 1	7.13	** < 0.001**	2.23	**0.012**	0.27
Total * Seascape 15	0.49	1	7.65	** < 0.001**	2.19	**0.009**	0.27
Snails * Seascape 3	0.64	− 1	6.29	** < 0.001**	15.62	** < 0.001**	0.71
Snails * Seascape 5	0.64	− 1	5.98	** < 0.001**	14.54	** < 0.001**	0.72
Snails * Seascape 15	0.41	+ 1	6.23	** < 0.001**	14.67	** < 0.001**	0.73
Crab/Shrimp * Seascape 27	0.44	− 1	4.39	**0.0003**	0.959	**0.039**	0.15
Bivalve * Seascape 13	0.62	+ 2	7.11	** < 0.001**	56.85	** < 0.001**	0.91
Richness * Seascape 21	− 0.45	0	7.35	** < 0.001**	3.00	**0.027**	0.40
Abundance * SST							
Total	0.41	0	6.74	** < 0.001**	0.81	**0.0504**	0.13
Total	0.42	− 1	6.43	** < 0.001**	0.61	**0.0781**	0.10
Snails	0.01	0	3.20	**0.0041**	0.00	**1.000**	< 0.01
Crab/Shrimp	0.44	0	4.36	**0.0002**	1.03	**0.0349**	0.15
Bivalve	− 0.08	0	2.09	**0.0479**	0.00	**0.6900**	< 0.01
Richness * SST	0.25	0	14.76	** < 0.001**	1.31	**0.0645**	0.16

r is the correlation coefficient; lag is the time lag (months); t and F for statistic, R-sq for adjusted R-squared, and D.Exp for % deviance explained by the model. Significant results (p < 0.05) are in bold. See Supplementary Table S7 in Supplementary material for full CCA results.

Seascape multivariate patterns explained 51 to 21% of the differences in taxa abundance and taxonomic composition of recruits (F = 2.64, p = 0.004, see Supplementary Table S11 and Fig. S5). Polychaetes, holothurians, gastropods (*Eulithidium affine*, *Bittiolum varium*, and limpets), bivalves (*Mytilaster solisianus*, Mytilidea), and brachyurans (Paguroidea and Penaeidae) were highly associated with Seascape classes (see Supplementary Fig. S5). The CAP ordination showed that recruit abundances from most spring months were relatively more similar, contained lower contributions of polychaetes and holothurians, and coincided with Warm, Blooms, High Nutrients Seascapes (see Supplementary Fig. S5). Other Seascape classes were not correlated with variations in recruit assemblage composition (see Supplementary Table S11).

### Modeling and validation

**Figure 3:**
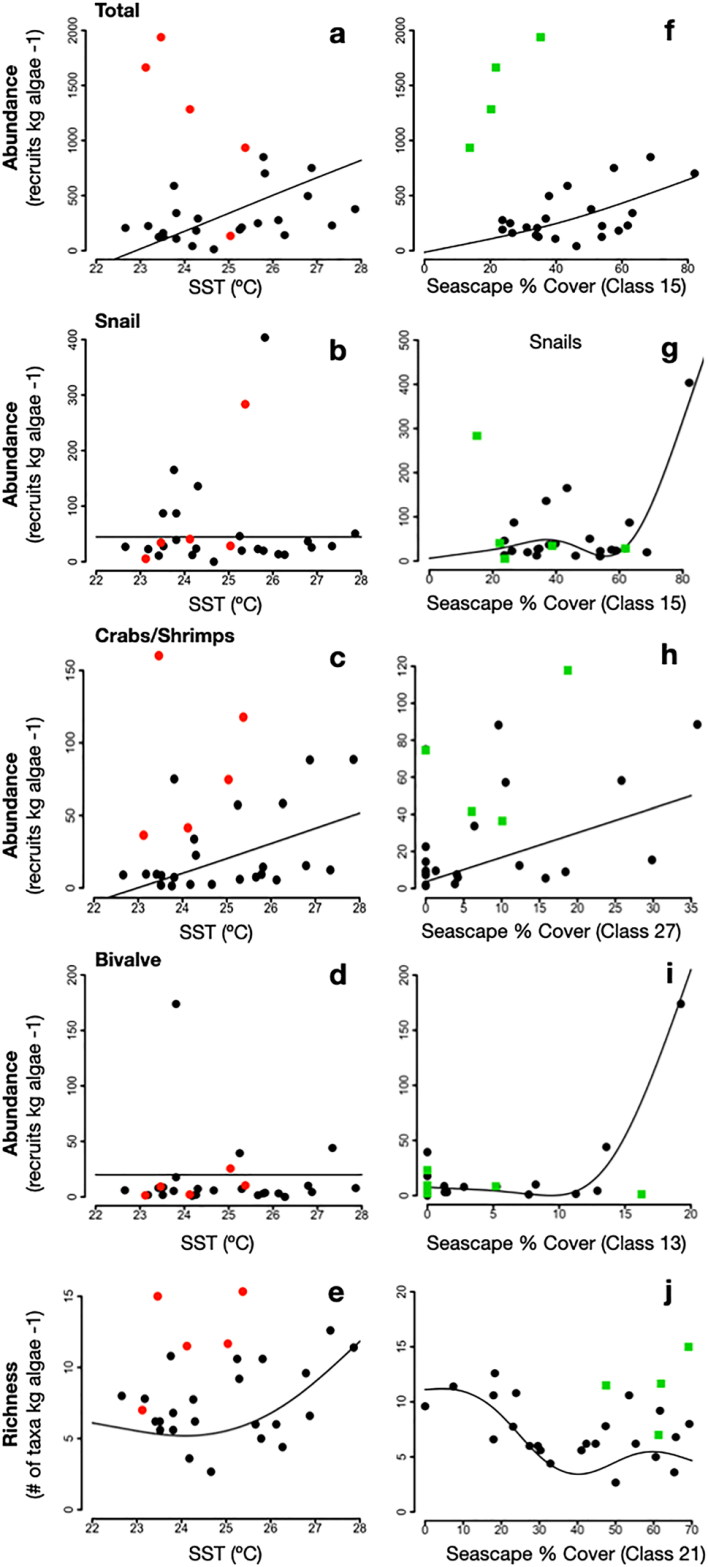
Results of the generalized linear models to examine SST (**a**–**e**) and local Seascape % cover (**f**–**j**) as predictors of temporal changes in recruitment (black dots, May 2017 to April 2019) and the observed patterns for the following months (red dots and green squares, July to November 2019).

Overall, the generalized additive models described well the associations between Seascape classes or SST and benthic recruit abundance and richness (Fig. 3; Table 3). According to these models, Seascape classes showed stronger correlations with recruitment than SST, with less deviance from predicting model (p < 0.05, Table 3). Higher model fitness was observed for snails (> 70%) and bivalves (> 90%) in association with the predominance of Tropical Seas and Subtropical Gyre Mesoscale Influenced seascapes, respectively. Validation with data from additional monitoring months showed similar trends to model predictions for richness and abundances of snails, crabs and shrimps, and bivalves (Fig. 3). This data was within the spectrum of variability from previous months, with the exception of total abundances which were much higher.

### Historical local Seascape patterns

**Figure 4:**
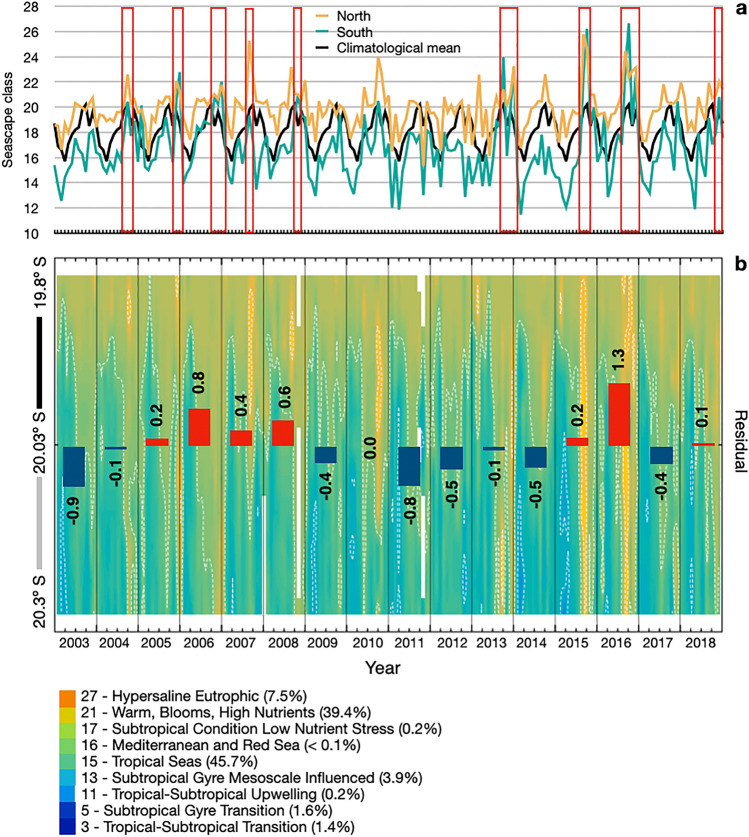
Monthly variations in Seascape classes in the study site (local scale ~ 30 km coastline, 465 km^2^) during 16 years before the recruitment samplings (January 2003 to December 2018). Differences in mean Seascape conditions were compared between the northern and southern areas of the study site, which exhibited distinct Seascape patterns during recruitment samplings (upper graph, (**a**), assessing the contribution of each Seascape class to the mean latitudinal variation (lower graph, (**b**), and quantifying the variation from the climatological mean (lower graph, **b**). Potential periods of reduced recruitment, when regional Seascapes are mostly dominated by warm high nutrient pelagic conditions, are highlighted (red squares; **a**,**b**). Note: line plots represent mean Seascape classes (upper graph, **a**); bars represent Seascape class residuals (lower graph, **b**).

During the years 2003 to 2019, nine Seascape classes were registered in the study region, characterizing both subtropical and tropical pelagic conditions (Fig. 4). Two Seascape classes were registered more frequently but differed in their spatial influence within the study region. Warm, Blooms, High Nutrients had a mean spatial coverage of 39% and predominated in the northern section (North of 20.03°S), whereas Tropical Seas was predominant in the southern section (South of 20.03°S) with a mean spatial cover of 46%. Both the average class and spatial coverage varied significantly between years, months, and seasons (p < 0.05, Table 1). Seascapes were highly seasonal with a higher cover of subtropical seascapes during summer and fall, whereas tropical nutrient-rich conditions dominated during late winter and spring (p = 0.01, see Supplementary Table S3; Fig. 4). Seascapes patterns were historically distinct in 2015 and 2016 (p < 0.05, see Supplementary Table S3), with increased spatial coverage of tropical waters along the northern and southern sections of the study area (Fig. 4).

The historical seascape database showed seven periods of potentially reduced benthic recruitment richness that were potentially associated with the dominance of Warm, Blooms, High Nutrients Seascape (class 21) over the study area. During the years 2005, 2006, 2007, 2008, 2013, 2015, 2016, and 2018, the Seascape class 21 exceeded the historical monthly mean. These periods coincided with the summer season and lasted from 2 to 6 months, being the longest in 2015 and 2016.

## Discussion

This is the first description and analysis of the MBON Pelagic Seascape Habitat Classification for the Southern Atlantic on the Brazilian coast (20.3º to 19.8º S), revealing that warm and tropical Seascape classes are the dominant pelagic conditions in the region (Figs. 1 and 4). As expected, we found a marked seasonal and local variability in Seascape classes, with Hypersaline eutrophic Seascapes occurring in the northern area during spring and summer (Figs. 1 and 4). In the southern area, episodic subtropical and deep-water intrusions occur during the fall in association with occasional upwelling events (Figs. 1 and 4). The dominant Seascapes represent pelagic conditions of tropical temperatures (22.5 to 26 °C), high salinity (34.5 to 38.1), low sea surface wave heights (< 0.7 m), and chlorophyll-a concentrations ranging from 0.07 to 2.09 mg m^−3^ (Figs. 1 and 4). The oceanographic attributes and ranges of the MBON Seascape classification agree with conditions described by previous surveys along this coast^48^. The spatial Seascape patterns in the study region revealed dynamic oceanographic conditions in the short (monthly) and in the long-term (annually), and the presence of different pelagic habitats within one single ecoregion^49^.

We observed that the dominant Seascapes on the Eastern Brazil Marine Ecoregion correlated with patterns of larval recruitment of reef benthic species at multiple temporal scales, corroborating our initial hypothesis that recruitment rates (i.e., abundance) and taxonomic composition would be highly correlated to changes in Seascapes through time, reflecting both local and regional variability in pelagic habitats (Fig. 3, supplementary material Fig. S5). This finding supports the interdependence of pelagic larval development and pelagic conditions such as water temperatures, food supply, and current transport in marine ecosystems^50,51^. Although these relationships have long been demonstrated for a range of coastal marine ecosystems, this study suggests that the MBON Seascape classification may capture the dynamic nature of coastal pelagic oceanography and partially explain variations in larval recruitment across reef habitats in the South Atlantic at multiple spatial and temporal scales. One improvement of using this Seascape assessment compared to single-variable analysis of pelagic conditions or traditional water mass classification (based on salinity and temperature) is precisely its set of attributes. MBON Seascapes deliver information about both physical and biochemical conditions influencing these invertebrate and provide insights into biological and ecological processes driven variability in benthic recruitment.

Recruitment of marine invertebrates is an ecological process that is under monitored at most habitats along the Brazilian coast and the data presented here is one of the longest recruitment time series published for the coastal reefs of the southwest Atlantic. These recruitment trends revealed high temporal variability what is commonly expected for benthic larval dynamics, and marked changes in taxonomic composition within the recruit assemblage probably following variations in settlement pools of different larval species (Fig. 2, Table 2, supplementary material Fig. S3^52^). We observed that gastropods, polychaetes, crustaceans, and holothurians dominate the recruitment assemblages, whose adults are also found in large abundances on these reefs^40,41^. The slight increase in both richness and diversity of recruits throughout the monitoring window (Fig. 2) suggests that the environmental conditions favored different recruits to settle and concentrate in large numbers in these macroalgal beds. Although benthic invertebrate recruitment is driven by a number of different factors such as the increase in reproduction rates^52^, it is highly affected by the availability of favorable settlement habitats^53^. *Sargassum* beds are shrinking at several sites in Brazil^54^, including the study region during these samplings (pers. obs.), which suggests that there could be less favoable habitats for these planktonic larvae to settle, and therefore, recruitment rates may increase at the remaining beds at first^53^.

Seascape patterns provided several insights about potential larval supply mechanisms in the studied tropical rocky reef communities. The Seascape patterns showed a lower recruitment rate in rocky reefs during warmer and higher nutrient conditions, which suggests that expected warmer temperatures would decrease recruitment success in this region^39,40^. Seascape analysis also indicates that intrusions of meteorological cold fronts, observed in this region mostly during fall and winter months^39,55^, change the coastal oceanography to subtropical conditions and likely promote rapid increases in recruitment through increased larval supply. According to these results, monthly benthic recruitment patterns at these macroalgae beds could be predicted by modeling seasonal variability in specific Seascapes classes (Fig. 3, supplementary material Fig. S5). Pelagic seasonality has been pointed out by several studies as an efficient tool to track variations in larval dynamics and other ecological processes at onshore benthic habitats [e.g.,^56–58^], which agree with the potential of Seascapes to predict marine community dynamics. We found that the relationship between Seascape dynamics and recruitment was taxon-specific (Fig. 3), revealing a range of reproductive strategies and larval dynamics in this area. Snails recruited preferentially with the onset of subtropical conditions, whereas Annelid worms recruited more during typical warm conditions of summer months. The spatial–temporal association between Seascape classes and recruitment also suggests that larval sources in the region may be originated from distant areas based on the result that larval recruitment was correlated with regional seascape dynamics. These findings have important implications for the management of coastal reef ecosystems as they could indicate that local assemblages are dependent on reproductive populations that are outside the managed boundaries of this MPA and that external larval inputs could be a strong driver of benthic biodiversity^60,61,69^.

Understanding changes in past, current, and future oceanographic conditions can help society prepare for and prevent further declines in marine biodiversity^1–4^. The analyses of pelagic Seascape dynamics from 2003 to 2019 provided us information about how often the different Seascape classes occur and persist in this region over the years, as well as what are the latitudinal extent of their influence (Fig. 4). We used these trends to identify several periods when warm, blooms and high nutrient waters covered most of the study region (60–100%) for 2 to 6 continuous months and resembled current (2017–2019) Seascape patterns when low recruitment events occurred. These years coincide with drier weather and warmer ocean conditions, such as the 2014–2016 El-Niño^62,63^, and could be related to lower recruitment seasons according to our findings. Thus, if these warm events become more frequent and with prolonged duration (e.g., heatwaves^64,65^), we should expect declines in benthic recruitment and significant implications for the long-term health of these reefs.

Considering that this pelagic Seascape classification method is primarily derived from remote sensing measurements, it is important to be aware of the limitations in analyzing nearshore areas. For instance, river plumes in this region may add confounding effects to satellite images and overestimate the discrimination of warm blooms^66,67^. Likewise, monthly Seascapes may not detect short-term key larval transport features (e.g. upwellings^30^), which could account for additional variability in recruitment patterns and associations with Seascapes. We shall also consider 2-yr duration of the study and the limited spatial coverage, which are fundamental aspects to be improved as Seascapes are applied to spatial planning and regional forecasting. Seasonality components in both recruitment and Seascapes could also be a confounding effect overestimating correlations that should be better explored when a longer time series is available^68^. Although Sargassum beds cover vast areas of the reef fringe and tidal flat and we had a high taxonomic range, the data presented in this manuscript obviously does not represent recruitment rates for all reef benthic invertebrate species. Many other invertebrates that are found in and on the reef rocks and sediments were not sampled by our monitoring, because they use different environments to settle^52^. Thus, expanding biodiversity monitoring range could provide new insights on the association between reef ecological patterns and seascape dynamics. One major advantage of using this Seascape classification is saving time on acquiring and processing large amounts of data from single oceanographic variables, and then developing tools to integrate them. Having one habitat categorization system that translates pelagic dynamics and efficiently associates with ecological processes will have multiple uses for science and conservation of marine ecosystems.

Our study supports that Seascape ecology has a promising potential to improve the understanding of the long-term dynamics of coastal marine ecosystems. Seascapes provide a more holistic picture of the pelagic system than single-variable classification (i.e. based only in SST) and modeling of larval dynamics, which is a common approach in larval ecology (e.g.,^28,69^). MBON Seascapes offer many advantages in identifying past and current changes to marine ecosystems and have key applications in marine spatial planning in the search for climate-resilient protected areas. The historical assessment and cross-correlation of Seascape classification with current biological dynamics is also a valuable tool to model past changes in marine ecosystems and evaluate their ecological dynamics across broader spatial–temporal scales. In addition, Seascapes may prove valuable to evaluate variability in the essential ocean variables EOVs (e.g. invertebrate richness and abundance,^70^), and may aid the process of identifying temporal responses of marine ecosystems on a changing climate^71^. With the challenges ahead, Seascapes classification requires reasonable technical training with biological data acquisition, which could facilitate its application within marine observation networks.

## Materials and methods

### Study area and sampling

This study was carried out at a coastal reef located inside two marine protected areas in the Eastern Brazil Marine Ecoregion (Refúgio da Vida Silvestre de Santa Cruz and Área de Proteção Costa das Algas; environmental permit by Instituto Chico Mendes #57819-1; see Supplementary Fig. S1). This is a tropical region with an average air temperature of 25 °C and sea surface temperature of 26 °C that has experienced significant warming trends during the last four decades (increase of 0.1 °C per 10 years and yearly anomalies reaching + 1 °C in the last two decades)^40,42^. Coastal oceanographic conditions are typically influenced by E-NE winds from the South Atlantic high-pressure system, strong internal tidal currents, and E-SE wave swells^55,72^. Meteorological cold fronts occur periodically and influence the vertical mixing of the water column and wave action on the coast^55^. Episodic upwelling events occur mostly during spring and summer^72^.

Our study site was located at a reef area that has been monitored by the Brazilian LTER site Coastal Habitats of Espírito Santo (HCES). The reefs in this region are mainly composed of ferruginous sandstone with small portions of biogenic calcareous (laterites), forming a very heterogeneous substrate with various shapes covered with dense and rich macroalgal beds^39,40^. Laterites occupy large littoral areas (tens of kilometers alongshore) extending from the upper intertidal zone to approximately 15–20 m deep and about 5 km offshore (Fig. 1). Benthic invertebrate larval recruitment was monitored for 24 months (May 2017 to November 2019; Supplementary Table S1;^39^). Invertebrate recruitment was measured in macroalgal beds (*Sargassum* sp.), natural substrates in the region commonly present in the upper subtidal zone along the reef fringe and inside tidal pools within the reef flat. Benthic invertebrate recruits were sampled monthly at the same location, where five samples (replicates) of *Sargassum* fronds with multiple branches (approximately 300 g of algae each) were manually collected during spring low tides. The sampling protocol was developed based on previous studies that investigated *Sargassum* associated fauna (e.g.^73^), which allowed collecting very abundant and rich assemblages of invertebrate recruits from resident and non-resident species (average 80 and maxima > 1000 individuals per sample). Samples were placed in plastic bags, taken to the laboratory, and frozen at − 20 °C for at least 24 h, then washed with freshwater on a 100 μm sieve to collect the fauna (e.g.^74,75^). All recruits (postlarvae, settlers, and first juveniles) were sorted, counted, and identified to the lowest possible taxonomic level under a stereomicroscope. For this study, we considered post-larvae as the planktonic or benthic larval form that are capable of settling, settlers as metamorphosed larval stages that hold specific morphological structures to live in the benthos, and first juveniles as small-sized individuals that are similar to the adult forms and may or not be able to reproduce;^75,76^). Discrimination between recruits and adults was carried out based on the size (adult average size for the species or group) and/or morphological characteristics particular of invertebrate post-larval and settlement phases (e.g.,^77^). Recruit morphological differences included the presence of eye-spot and shell morphology for bivalves and gastropods, number and size of body segments for polychaetes, body morphology for cnidarians, echinoderms, barnacles, sponges, and tunicates, abdomen attachment for crabs, and shell thickness for polyplacophorans (See Supplementary Table S2).

### Seascape characterization

Oceanographic conditions during the sampling window (May 2017 to April 2019, July to November 2019) and in the long-term (2003 to 2019, years available in the database) were characterized synoptically through variations in MBON Seascape Pelagic Habitat Classification^35,36^. Seascape categories (a total of 32 classes) are derived from dynamic fields of satellite and modeled data, defined according to sea surface temperature (SST), sea surface salinity (SSS), absolute dynamic topography (ADT), chromophoric dissolved organic material (CDOM), surface chlorophyll-a (CHLA), and normalized fluorescent line height (NFLH). The dataset is available from the NOAA Coast and OceanWatch Programs, on a monthly frequency and along a 5 km^2^ grid^35,36^. Seascape classes were characterized monthly along the marine protected area (local scale ~ 30 km coastline, 465 km^2^, Long. − 40.3º to − 39.8º W, Lat. 20.3º to 19.8º S) and the region (approximately 700 km coastline, 357,500 km^2^), Long. − 42.5º to − 36.0º W, Lat. 16.5º to 22.5º S; Fig. 1). For the long-term approach, only local Seascape patterns (~ 30 km coastline, Lat. 20.3º to 19.8º S, longitudinal averaged) were included and the northern and southern portions were examined separately (Lat 19.8º to 20.02ºS averages for North, Lat 20.03 to 20.3ºS averages for South).

Seascape SSTs were compared to in situ temperature using loggers placed on the reef rocks at the lower intertidal zone at the study site (Dec-2017 to Jun-2018 by HOBO Loggers U24, Onset®; Jun-2019 to Apr-2020 by EnvLoggers, Electric Blue®). Seascape SSTs were positively correlated with in situ SST measurements (df = 4, t = 2.85, r = 0.81, p = 0.0459) and an average of 1.65 °C lower. Calibration of the full Seascape variables was not possible due to the lack of oceanographic data.

Additionally, we obtained daily SST measurements from the NOAA High-resolution Blended Analysis of Daily SST^78^ for the study area (local scale ~ 30 km coastline, 465 km^2^; Long. − 40.3º to − 39.8º W, Lat. 20.3º to 19.8º S) during the study period. These measurements were used to calculate the average SST for each sampling month and were included in the analysis comparing Seascapes and SST efficiency to predict recruitment patterns.

Seascape and SST data were downloaded from the NOAA ERDDAP data server^79^, through the following addresses: https://cwcgom.aoml.noaa.gov/erddap/griddap/noaa_aoml_4729_9ee6_ab54.html. ^35,36^ https://upwell.pfeg.noaa.gov/erddap/griddap/noaa_psl_a0d9_5e0c_6128.html. ^78^

### Data analysis

Seascape monthly patterns were described using (1) differences in the Seascape % Cover (relative and absolute), calculated by the number of pixels of each Seascape class occupying the study region per month, (2) variations in the mean Seascape class, the quantitative value from averaging all class values available for the study site (local scale, average n = 76 pixels) and region (average n = 9800 pixels), and (3) seasonal Seascape maps to illustrate differences at the regional scale. Month, seasonal, and year differences in Seascapes within the study region were evaluated using 3-way permutational multivariate analysis of variance (PERMANOVA;^80^), using distance matrices of relative % cover of Seascape classes during the sampling window (2017–2019) and in the long-term (2003–2019). PERMANOVAs designs tested year (factor 1) fixed with 2 levels (2017 to 2018, 2018 to 2019), seasons (factor 2) fixed with 4 levels (fall, winter, spring, summer), and months (factor 3) random with 3 levels (3 months in each season), and were complemented by post-hoc all pair-wise comparisons. Local (~ 30 km coastline, 465 km^2^) and regional (700 km coastline, 357,500 km^2^) Seascape trends were compared by Pearson correlation^81^, using the monthly averages of the class values (0 to 33;^35,36^).

Recruitment patterns were determined from the total and relative abundance of recruits (recruits per kg of algae), recruit diversity (Shannon–Wiener index), and richness (S) in each period sampled. Representativeness of samplings and low sample size were evaluated through taxa accumulation and rarefaction curves for the monitored years (2017–2018, and 2018–2019;^82–84^). Variability in recruitment between year, seasons, and months was tested by 3-way ANOVAs (ANOVA, with unbalanced replicates;^81^), complemented by a PERMANOVA to assess differences in recruit assemblages (taxonomic composition). Both ANOVAs and PERMANOVA followed the same design described above for the Seascape evaluation, followed by Tukey HSD and PERMANOVA post-hoc pairwise tests. The long-term trend in recruitment was assessed using regression analyses adjusted to the diversity and richness time series^81^.

The association between recruitment patterns (abundance, diversity, and richness) and Seascapes classes was assessed by cross-correlation analyses. These analyses were carried out using the average month values for each ecological parameter and the relative percentage cover of each seascape class in these months. Correlations were considered significant when p < 0.01 and lags were between 0 to 2 months, based on the average development time for tropical coastal invertebrate larvae (weeks to months^81^). Additionally, a canonical analysis of principal coordinates (CAP;^85,86^) was carried out to identify the class or set of classes with a higher potential to drive changes in recruit assemblages. CAP was performed comparing variations in recruit abundances for each taxon and the frequency of occurrence of each Seascape class.

Generalized linear models (GAM;^87,88^) were applied to the dataset (May 2017 to April 2019) to examine Seascapes and SST as predictors of temporal changes in recruitment. The correlation coefficient, adjusted R-squared, and % deviance were used as parameters to evaluate model fitness and the levels of association between variables. The observed patterns for the following months (July to November 2019) were graphically compared to GAM trends. Only Seascapes that were significantly correlated to recruitment and corresponding lags were included in this analysis.

Long-term analyses of Seascape variability included: (1) graphical assessment of the monthly variations of Seascape classes along the study region; (2) estimation of average Seascape classes for the northern and southern part of the study region, using numerical Seascape classification to perform zonal averages for the available grid points North to the sampling site n = 5, and South to the sampling site n = 6); (3) calculation of the Seascape climatology (Jan to Dec) by averaging numerical classes from the 16 years available in the dataset for each month; (4) estimations of the residuals, the differences between observed Seascape numerical classes and climatological means; (5) identification local increase in the cover of Warm, Blooms, High Nutrients Seascape, Seascape conditions for potentially reduced recruitment, according to our results.

Data were Log x + 1 or square-root transformed when needed to fit the assumptions of ANOVAs, correlation analysis, and CAP (normality and homogeneity of variances), verified by Kolmogorov–Smirnov and Cochran tests; and square-root x + 1 prior to PERMANOVAs. We assumed α = 0.05 and determined the significant p values using the Benjamin-Hochberg false discovery rate method^89,90^. Graphical and analytical processing were performed in Panoply 4.8.1^91^ for Seascape visualization and extraction, Numbers (Apple Inc.) for charts, and R project^92^ for statistics (R packages ‘stats’ for general calculations, ‘GAD’^93^ and ‘outliers’^94^ for ANOVAs and post-hoc tests, ’vegan'^95^ for PERMANOVA, ’rich’^96^ for ecologic indexes, and 'gam' for modeling^97^.

## Supplementary Information

Supplementary Information.

## Data Availability

LTER recruitment data is available at the Ocean Biodiversity Information System (OBIS) repository http://doi.org/10.25607/rtgvpa; and will be updated with the data in this manuscript after publishing.
