# Supplementary material for: Reef larval recruitment in response to seascape dynamics in the SW Atlantic
Source: Sci Rep. 2022 May 11;12:7750. doi: 10.1038/s41598-022-11809-1 (PMC9095688; doi:10.1038/s41598-022-11809-1)
Supplement: Supplementary file 1 — Supplementary Information. [file 41598_2022_11809_MOESM1_ESM.pdf]

**Supplementary Material**

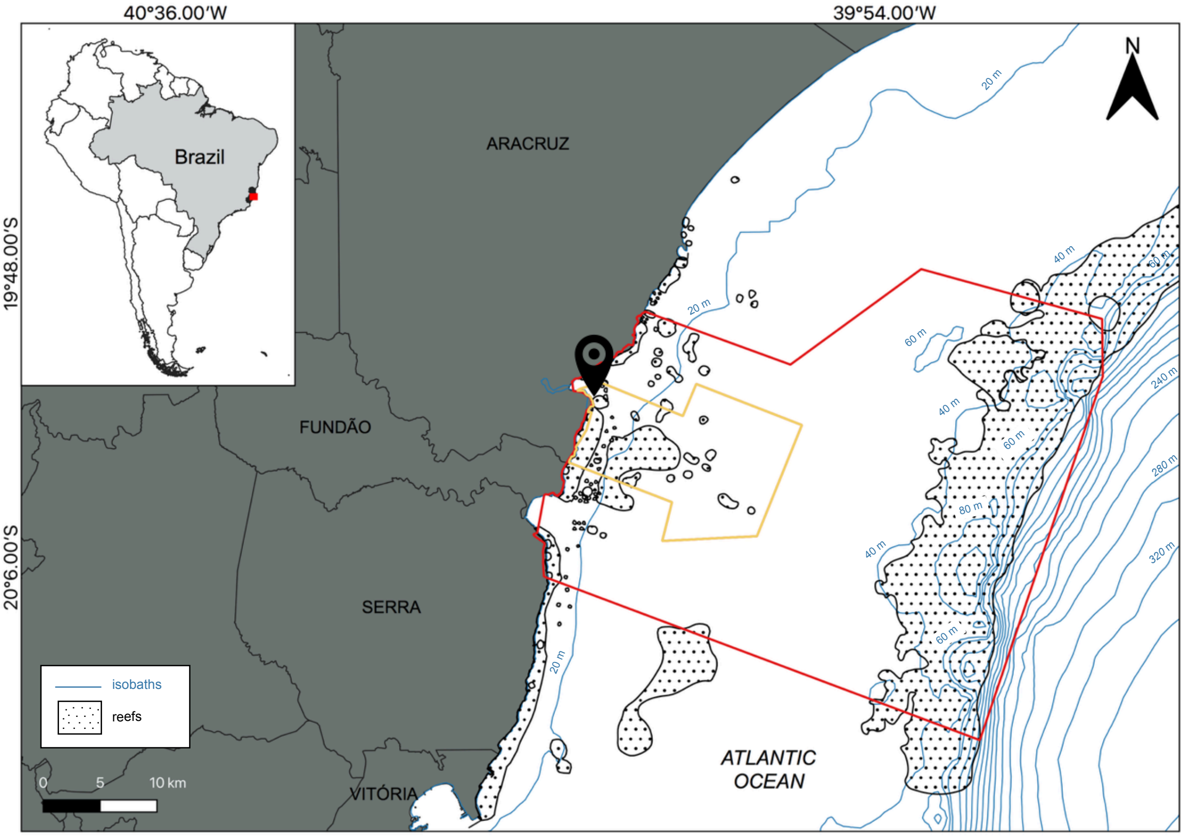


Figure S1. Location of the LTER HCES site (black symbol) in the Costa das Algas Marine Protection Area (red line) and Santa Cruz Wildlife Refuge (yellow line), on the Eastern coast of Brazil. Macroalgal reefs are highlighted by the dotted areas. Blue lines represent isobaths of 20 m intervals.

Table S1. List of the sampling dates from May 2017 to April 2019.

| | 2017-2018 | 2018-2019 | 2019-2020 |
|---|---|---|---|
| May | 25/05/17 | 16/05/18 | 06/0719 |
| Jun | 27/06/17 | 14/06/18 | 31/08/19 |
| Jul | 23/07/17 | 27/07/18 | 28/09/19 |
| Aug | 22/08/17 | 31/08/18 | 29/10/19 |
| Sep | 20/09/17 | 26/09/18 | 24/11/19 |
| Oct | 19/10/17 | 25/10/18 | |
| Nov | 16/11/17 | 28/11/18 | |
| Dec | 18/12/18 | - | |
| Jan | 16/01/18 | 16/01/19 | |
| Feb | 16/02/18 | 19/02/19 | |
| Mar | 17/03/18 | 21/03/19 | |
| Apr | 17/04/18 | 20/04/19 | |

Table S2. List of the consulted references and sites used for recruit identification and size discrimination (post-larvae, settler, and first juvenile).

| | Reference and sites |
|---|---|
| General | Strathmann, M. (1987). Reproduction and Development of Marine Invertebrates of the Northern Pacific Coast: Data and Methods for the Study of Eggs, Embryos, and Larvae. Seattle; London: University of Washington Press. http://www.jstor.org/stable/j.ctvcwnh8b<br>McEdward, L. (Ed.). (1995). Ecology of Marine Invertebrate Larvae (1st ed.). CRC Press. https://doi.org/10.1201/9780138758950<br>Shanks, A.L. (editor). 2001. An Identification Guide to the Larval Marine Invertebrates of the Pacific Northwest. Oregon State University Press, Corvallis, Oregon. 320 pages. ISBN 0-87071-531-3.<br>Carrier, T., Reitzel, A., Heyland, A. 2018. Evolutionary Ecology of Marine Invertebrate Larvae. Oxford Scholarship Online. DOI: 10.1093/oso/9780198786962.001.0001<br><br>World Register of Marine Species https://www.marinespecies.org/<br>Marine Species Identification Portal http://species-identification.org/<br>SeaLifeBase https://www.sealifebase.ca/ |
| Specific to | |
| Bivalves | Loosanoff, V.L., Davis, H.C. 1963. Rearing of Bivalve Mollusks. Advances in Marine Biology, 1: 1-136. DOI: 10.1016/S0065-2881(08)60257-6.<br>Le Pennec, M. (1980). The larval and post-larval hinge of some families of bivalve molluscs. Journal of the Marine Biological Association of the United Kingdom, 60(3), 601-617. doi:10.1017/S0025315400040297<br>Fuller, S. C., & Lutz, R. A. (1989). Shell morphology of larval and post-larval mytilids from the north-western Atlantic. Journal of the Marine Biological Association of the United Kingdom, 69(01), 181. doi:10.1017/s0025315400049183 |
| Gastropods (Snails and limpets) | Lima, G. M., & Lutz, R. A. (1990). The relationship of larval shell morphology to mode of development in marine prosobranch gastropods. Journal of the Marine Biological Association of the United Kingdom, 70(03), 611. doi:10.1017/s0025315400036626<br>Longo, P.A.S., Fernandes, M.C., Leite, F.P.P., Passos, F.D. Gastropoda (Mollusca) associados a bancos de Sargassum sp. no Canal de Sãoo Sebastião – São Paulo, Brasil. Biota Neotropica. 14(4): e20140115. http://dx.doi.org/10.1590/1676-06032014011514 |
| Polychaetes | Blake, J. (1975). The Larval Development of Polychaeta from the Northern California Coast. I. Cirriformia spirabrancha (Family Cirratulidae). Transactions of the American Microscopical Society, 94(2), 179-188. doi:10.2307/3224978<br>Giangrande, A. (1997). Polychaete reproductive patterns, life cycle and life histories: an overview. Oceanogr. Mar. Biol. Ann. Rev. 35: 323-386. |
| Cnidarians | Riemann-Zürneck, K. (1998) How Sessile are Sea Anemones? A Review of Free-living Forms in the Actiniaria Cnidaria: Anthozoa. Marine Ecology, 19: 247-261. https://doi.org/10.1111/j.1439-0485.1998.tb00466.x<br>Helm, R.R. (2018), Evolution and development of scyphozoan jellyfish. Biol Rev, 93: 1228-1250. https://doi.org/10.1111/brv.12393 |
| Echinoderms | George, S.B., Lawrence, J.M., Lawrence, A.L. 2004. Complete larval development of the sea urchin Lytechinus variegatus fed an artificial feed. Aquaculture, 242, Issues 1–4: 217-228. DOI: 10.1016/j.aquaculture.2004.06.024.<br>Huang, W., Huo, D., Yu, Z., Ren, C., Jiang, X., Luo, P., Chen, T., Hu, C. 2018. Spawning, larval development and juvenile growth of the tropical sea cucumber Holothuria leucospilota, Aquaculture, 488, 22-29. DOI:10.1016/j.aquaculture.2018.01.013.<br>Pawson, D.L., Pawson, D.J., King, R.A. (2010). A taxonomic guide to the Echinodermata of the South Atlantic Bight, USA: 1. Sea cucumbers (Echinodermata: Holothuroidea). Zootaxa, Vol. 2449 No. 1: 7. DOI: https://doi.org/10.11646/zootaxa.2449.1.1 |

| | |
|---|---|
| Barnacles | Lang, W. H. 1979. Larval development of shallow water barnacles of the Carolinas (Cirripedia: Thoracica) with keys to naupliar stages. Technical Reports, U.S. Department of Commerce, National Oceanic and Atmospheric Administration, National Marine Fisheries Services, Circular 421. p. 47 |
| Sponges | Maldonado, M. 2006. The ecology of the sponge larva. Canadian Journal of Zoology. 84(2): 175-194. https://doi.org/10.1139/z05-177 |
| Tunicates | Cloney, R.A. 1982. Ascidian Larvae and the Events of Metamorphosis. American Zoologist, 2(4): 817–826. DOI: 10.1093/icb/22.4.817 |
| Crabs and shrimps | Schram, F., Klein, C.V. 2012. Treatise on Zoology - Anatomy, Taxonomy, Biology. The Crustacea, Volume 9 Part B Decapoda: Astacidea P.P. (Enoplometopoidea, Nephropoidea), Glypheidea, Axiidea, Gebiidea, and Anomura. BRILL, Leiden. ISBN: 978-90-47-43017-9 |
| Polyplacophorans | Kniprath, E. Ontogenetic plate and plate field development in two chitons,Middendorffia andIschnochiton . Wilhelm Roux' Archiv 189, 97–106 (1980). https://doi.org/10.1007/BF00848498 |

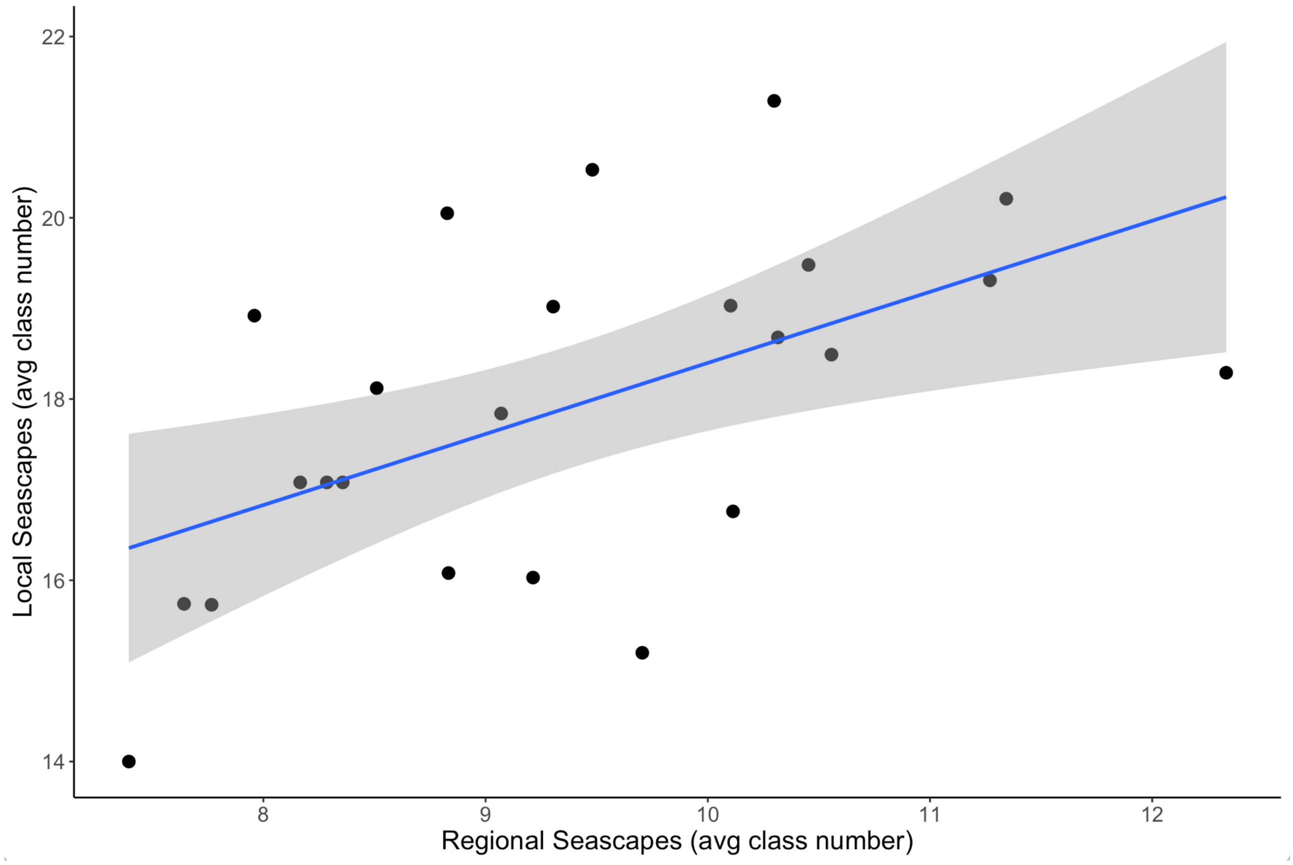


Figure S2. Comparison of local and regional variations in Seascape classes (average monthly values).

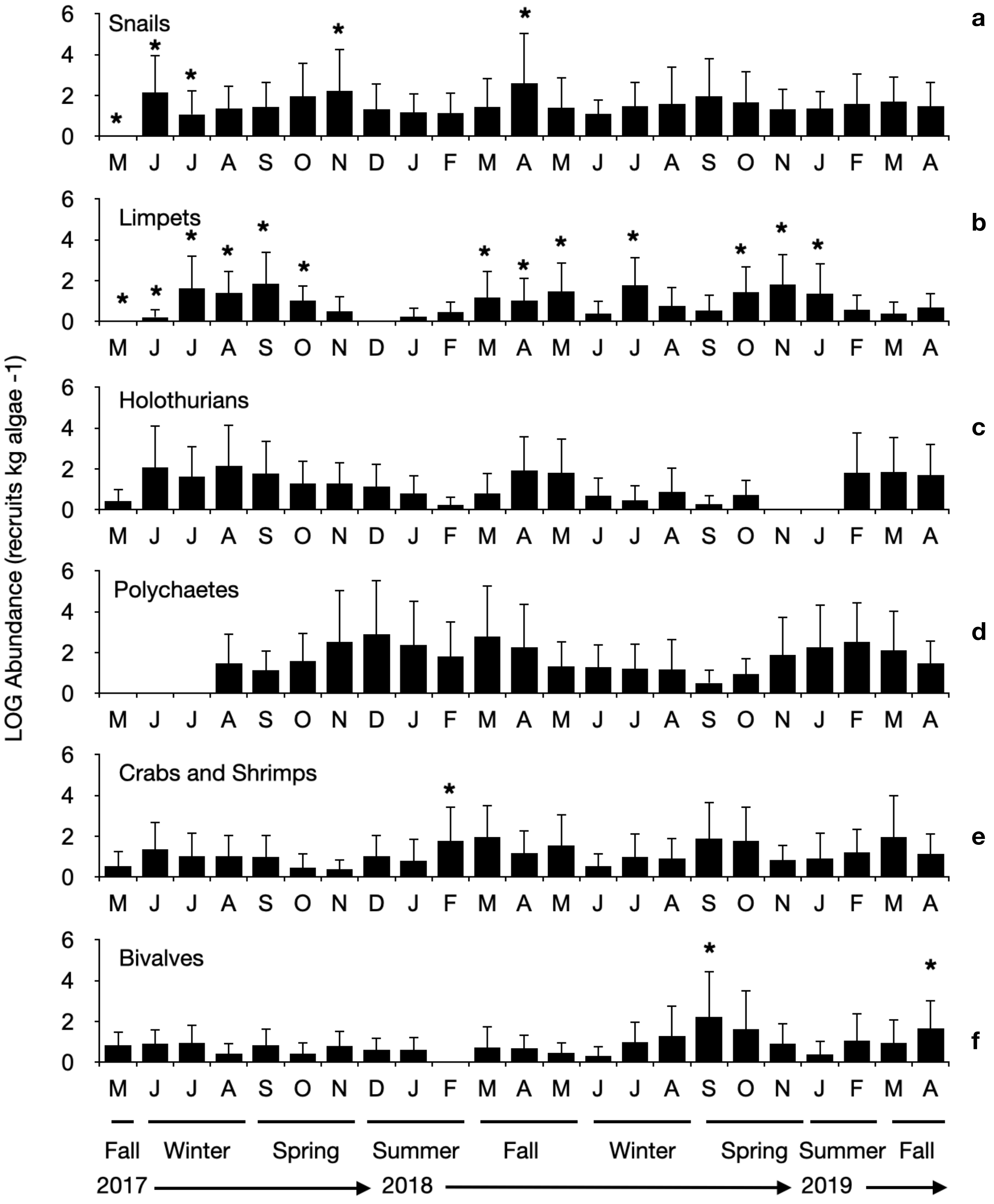


Figure S3. Monthly recruit assemblage abundances (number of recruits per taxon per kg of algae) at the study sites. Note: averages and standard deviations are represented by columns and error bars respectively.

Table S3. Significant results of pairwise PERMANOVA to assess differences in in the coverage of Seascape categories (number of pixels) between seasons and months during the study sampling 2017-2019 and in the previous 16 years (2003-2018).

**2017-2019**

| **Seasons** | ***p*** | **Months** | ***p*** | **Months** | ***p*** | **Months** | ***p*** |
|---|---|---|---|---|---|---|---|
| Fall vs. Summer | 0.014 | May-Jun | 0.333 | Jul-Oct | 1.000 | Oct-Nov | 1.000 |
| Fall vs. Spring | 0.013 | May-July | 0.333 | Jul-Nov | 1.000 | Oct-Dec | 0.333 |
| Fall vs. Winter | 0.011 | May-Aug | 0.666 | Jul-Dec | 0.333 | Oct-Jan | 0.333 |
| Summer vs. Winte | 0.016 | May-Sep | 0.333 | Jul-Jan | 0.333 | Oct-Feb | 0.333 |
| Spring vs. Winter | 0.032 | May-Oct | 0.333 | Jul-Feb | 0.333 | Oct-Mar | 0.333 |
| | | May-Nov | 0.666 | Jul-Mar | 0.333 | Oct-Apr | 0.333 |
| | | May-Dec | 0.333 | Jul-Apr | 0.333 | Nov-Dec | 0.333 |
| | | May-Jan | 0.333 | Aug-Sep | 0.666 | Nov-Jan | 0.666 |
| | | May-Feb | 0.333 | Aug-Oct | 0.666 | Nov-Feb | 0.333 |
| | | May-Mar | 0.666 | Aug-Nov | 0.666 | Nov-Mar | 1.000 |
| | | May-Apr | 0.666 | Aug-Dec | 0.333 | Nov-Apr | 0.333 |
| | | Jun-Aug | 1.000 | Aug-Jan | 0.333 | Dec-Jan | 0.333 |
| | | Jun-Sep | 0.666 | Aug-Feb | 0.666 | Dec-Feb | 0.333 |
| | | Jun-Oct | 0.666 | Aug-mar | 0.666 | Dec-Mar | 0.333 |
| | | Jun-Nov | 1.000 | Aug-Apr | 0.333 | Dec-Apr | 0.333 |
| | | Jun-Dec | 0.333 | Sep-Oct | 0.333 | Jan-Feb | 1.000 |
| | | Jun-Jan | 0.333 | Sep-Nov | 0.666 | Jan-Mar | 0.333 |
| | | Jun-Feb | 0.333 | Sep-Dec | 0.333 | Jan-Apr | 0.333 |
| | | Jun-Mar | 0.333 | Sep-Jan | 0.333 | Feb-Mar | 0.666 |
| | | Jun-Apr | 0.333 | Sep-Feb | 0.333 | Feb-Apr | 0.666 |
| | | Jul-Aug | 1.000 | Sep-Mar | 0.333 | Mar-Apr | 1.000 |
| | | Jul-Sep | 0.333 | Sep-Apr | 0.333 | | |

**2003-2018**

| **Years** | ***p*** | **Seasons** | ***p*** | **Months** | ***p*** | **Months** | ***p*** |
|---|---|---|---|---|---|---|---|
| 2003*2015 | 0.013 | Fall vs. Summer | 0.001 | Jan-Feb | 0.002 | Apr-Aug | 0.001 |
| 2003*2016 | 0.022 | Fall vs. Winter | 0.001 | Jan-Mar | 0.001 | Apr-Sep | 0.001 |
| 2004*2015 | 0.026 | Fall vs. Spring | 0.001 | Jan-Apr | 0.001 | Apr-Oct | 0.001 |
| 2009*2016 | 0.039 | Summer vs Winter | 0.001 | Jan-May | 0.004 | Apr-Nov | 0.001 |
| 2012*2016 | 0.027 | Summer vs. Spring | 0.001 | Jan-Jun | 0.008 | Apr-Dec | 0.001 |
| | | Winter vs. Spring | 0.001 | Jan-Jul | 0.003 | May-Jun | 0.038 |
| 2004*2016 | 0.068 | | | Jan-Aug | 0.003 | May-Jul | 0.002 |
| 2005*2016 | 0.099 | | | Feb-May | 0.050 | May-Aug | 0.001 |
| 2011*2016 | 0.070 | | | Feb-Jun | 0.001 | May-Sep | 0.001 |
| 2017*2016 | 0.092 | | | Feb-Jul | 0.001 | May-Oct | 0.001 |
| | | | | Feb-Aug | 0.001 | May-Nov | 0.002 |
| | | | | Feb-Sep | 0.001 | May-Dec | 0.003 |
| | | | | Feb-Oct | 0.001 | Jun-Sep | 0.001 |
| | | | | Feb-Nov | 0.001 | Jun-Oct | 0.001 |
| | | | | Feb-Dec | 0.001 | Jun-Nov | 0.009 |
| | | | | Mar-May | 0.001 | Jun-Dec | 0.012 |
| | | | | Mar-Jun | 0.001 | Jul-Sep | 0.001 |
| | | | | Mar-Jul | 0.001 | Jul-Oct | 0.001 |
| | | | | Mar-Aug | 0.001 | Jul-Nov | 0.010 |
| | | | | Mar-Sep | 0.001 | Jul-Dec | 0.017 |
| | | | | Mar-Oct | 0.001 | Aug-Sep | 0.013 |
| | | | | Mar-Nov | 0.001 | Aug-Oct | 0.00` |
| | | | | Mar-Dec | 0.001 | Aug-Nov | 0.016 |
| | | | | Apr-Jun | 0.001 | Aug-Dec | 0.040 |
| | | | | Apr-Jul | 0.001 | | |

Table S4. List of high taxonomic composition HTC of recruits sampled in macroalgal Sargassum beds during the study.

| Taxonomic group | HTC | LSID |
|---|---|---|
| Bivalvia | *Mytilaster solisianus* | urn:lsid:marinespecies.org:taxname:506154 |
| | *Lunarca ovalis* | urn:lsid:marinespecies.org:taxname:420721 |
| | *Isognomon bicolor* | urn:lsid:marinespecies.org:taxname:420737 |
| | *Pinctada imbricata* | urn:lsid:marinespecies.org:taxname:207901 |
| | Solecurtidae | urn:lsid:marinespecies.org:taxname:1784 |
| | Other Mytilidea | urn:lsid:marinespecies.org:taxname:211 |
| | Other Bivalvia | urn:lsid:marinespecies.org:taxname:105 |
| Cnidaria | Ephira | urn:lsid:marinespecies.org:taxname:1267 |
| | Actiniaria | urn:lsid:marinespecies.org:taxname:1360 |
| | Other Anthozoa | urn:lsid:marinespecies.org:taxname:1292 |
| Crustacea | Cirripedia | urn:lsid:marinespecies.org:taxname:1292 |
| | *Pachygrapsus transversus* | urn:lsid:marinespecies.org:taxname:107457 |
| | Xanthidae | urn:lsid:marinespecies.org:taxname:106769 |
| | Other Brachiurans | urn:lsid:marinespecies.org:taxname:106673 |
| | Paguroidea | urn:lsid:marinespecies.org:taxname:106687 |
| | Epialtidae | urn:lsid:marinespecies.org:taxname:196143 |
| | Penaeidae | urn:lsid:marinespecies.org:taxname:106727 |
| Echinodermata | Asteroidea | urn:lsid:marinespecies.org:taxname:123080 |
| | Crinoidea | urn:lsid:marinespecies.org:taxname:123081 |
| | Echinoidea | urn:lsid:marinespecies.org:taxname:123082 |
| | Holothuroidea | urn:lsid:marinespecies.org:taxname:123083 |
| | Ophiuroidea | urn:lsid:marinespecies.org:taxname:123084 |
| Gastropoda | *Aplysia* sp. | urn:lsid:marinespecies.org:taxname:137654 |
| | *Alaba incerta* | urn:lsid:marinespecies.org:taxname:419526 |
| | *Amphithalamus glabus* | urn:lsid:marinespecies.org:taxname:532382 |
| | *Anachis fenneli* | urn:lsid:marinespecies.org:taxname:511395 |
| | *Assiminea succinea* | urn:lsid:marinespecies.org:taxname:160168 |
| | *Astyris lunata* | urn:lsid:marinespecies.org:taxname:160102 |
| | *Bittiolum varium* | urn:lsid:marinespecies.org:taxname:160174 |
| | *Bonea jadisi* | urn:lsid:marinespecies.org:taxname:532447 |
| | *Caecum ryssotitum* | urn:lsid:marinespecies.org:taxname:419594 |
| | *Calliostoma* sp. | urn:lsid:marinespecies.org:taxname:138584 |
| | *Cerithium atratum* | urn:lsid:marinespecies.org:taxname:224572 |
| | *Chrysallida* sp. | urn:lsid:marinespecies.org:taxname:138401 |
| | *Lottia* sp. | urn:lsid:marinespecies.org:taxname:160336 |
| | *Engina turbinella* | urn:lsid:marinespecies.org:taxname:234108 |
| | *Eulithidium affine* | urn:lsid:marinespecies.org:taxname:419483 |
| | *Fissurella* sp. | urn:lsid:marinespecies.org:taxname:138013 |
| | *Lithopoma phoebium* | urn:lsid:marinespecies.org:taxname:413409 |
| | *Echinolittorina lineolata* | urn:lsid:marinespecies.org:taxname:446863 |
| | *Parvanachis obesa* | urn:lsid:marinespecies.org:taxname:160440 |
| | *Pyrunculus caelatus* | urn:lsid:marinespecies.org:taxname:581411 |
| | *Stramonita brasiliensis* | urn:lsid:marinespecies.org:taxname:574250 |
| | *Tegula viridula* | urn:lsid:marinespecies.org:taxname:413470 |
| | *Tenaturris fulgens* | urn:lsid:marinespecies.org:taxname:435029 |
| | *Turbonilla* sp. | urn:lsid:marinespecies.org:taxname:138421 |

| | | |
|---|---|---|
| | *Turbonilla multicostata* | urn:lsid:marinespecies.org:taxname:420491 |
| | *Volvarina* sp. | urn:lsid:marinespecies.org:taxname:138167 |
| | Other Cerithidea | urn:lsid:marinespecies.org:taxname:204619 |
| | Other Heterobranchia | urn:lsid:marinespecies.org:taxname:14712 |
| | Other Gastropods | urn:lsid:marinespecies.org:taxname:101 |
| Polyplacophora | | urn:lsid:marinespecies.org:taxname:55 |
| Polychaetes | Sabellidae | urn:lsid:marinespecies.org:taxname:985 |
| | Other Polychaetes | urn:lsid:marinespecies.org:taxname:883 |
| Porifera | | urn:lsid:marinespecies.org:taxname:558 |
| Sipuncula | | urn:lsid:marinespecies.org:taxname:1268 |
| Tunicata | Ascidiacea | urn:lsid:marinespecies.org:taxname:1839 |
| Unidentified taxa | about 10 speciemens | |

Table S5. Results of Tukey HSD pair-wise comparisons to assess differences in recruit assemblage abundances (total number of recruits), diversity (Shanon-Wiener Index), and richness (number of species per taxa) between seasons. Significant results ($p < 0.05$) are in bold.

| | ***p*** | | | | | |
|---|---|---|---|---|---|---|
| | **Abundance** | **Composition** | **Diversity** | **Richness** | | |
| Fall vs. Summer | 0.3742 | **0.006** | **0.0003** | **0.0178** | | |
| Fall vs. Spring | 0.9827 | **0.006** | 0.4748 | 0.9999 | | |
| Fall vs. Winter | 0.1017 | **0.006** | 0.9442 | **0.0025** | | |
| Summer vs. Spring | 0.5880 | **0.006** | **< 0.0001** | **0.0184** | | |
| Summer vs. Winter | **0.0012** | **0.006** | **0.0023** | 0.9685 | | |
| Spring vs. Winter | **0.0416** | **0.018** | 0.1935 | **0.0026** | | |
| | **Snail** | **Limpet** | **Holothuria** | **Polychaete** | **Crabs and Shrimps** | **Bivalves** |
| Fall vs. Summer | 0.8153 | 0.6848 | **0.0126** | **0.0004** | 0.2809 | **0.2809** |
| Fall vs. Spring | 0.1255 | **0.0450** | 0.0979 | 0.3257 | 0.2208 | 0.2208 |
| Fall vs. Winter | 0.9833 | 0.4305 | 0.7440 | **< 0.0001** | **0.0251** | 0.0251 |
| Summer vs. Spring | **0.0177** | **0.0023** | 0.8116 | **< 0.0001** | 0.9999 | **0.9999** |
| Summer vs. Winter | 0.9511 | 0.0570 | 0.1459 | **< 0.0001** | 0.7843 | **0.7843** |
| Spring vs. Winter | 0.0537 | 0.6706 | 0.5536 | **< 0.0080** | 0.7858 | **0.7858** |

Table S6. Significant results of Tukey HSD pair-wise comparisons to assess differences in recruit assemblage abundances (total number of recruits) and diversity (Shannon-Wiener) between months.

**Abundance**

| **Month** | ***p*** | **Month** | ***p*** | **Month** | ***p*** |
|---|---|---|---|---|---|
| 2017Aug-2017May | < 0.0001 | 2018Jun-2017Nov | < 0.0001 | 2018Jul-2018Apr | 0.0054 |
| 2017Jul-2017May | < 0.0001 | 2017Oct-2017Dec | 0.0034 | 2018Jun-2018Apr | < 0.0001 |
| 2017Jun-2017May | < 0.0001 | 2017Sep-2017Dec | 0.0285 | 2018May-2018Apr | 0.0459 |
| 2017Dec-2017May | < 0.0001 | 2017Jul-2017Dec | < 0.0001 | 2017Oct-2018Apr | 0.0449 |
| 2017Nov-2017May | < 0.0001 | 2018Aug-2017Dec | < 0.0001 | 2018Sep-2018Aug | 0.0188 |
| 2017Oct-2017May | < 0.0001 | 2018Feb-2017Dec | 0.0010 | 2019Mar-2018Aug | 0.0064 |
| 2017Sep-2017May | < 0.0001 | 2018Jul-2017Dec | 0.0002 | 2017Jun-2018Aug | 0.0500 |
| 2018Apr-2017May | < 0.0001 | 2018Jun-2017Dec | < 0.0001 | 2019Feb-2018Aug | 0.0002 |
| 2018Aug-2017May | 0.0016 | 2018May-2017Dec | 0.0035 | 2019Feb-2018Jul | 0.0394 |
| 2018Feb-2017May | < 0.0001 | 2018Nov-2017Dec | 0.0243 | 2019Feb-2017Jul | 0.0101 |
| 2018Jan-2017May | < 0.0001 | 2018Oct-2017Dec | 0.0095 | 2019Feb-2018Jun | < 0.0001 |
| 2018Jul-2017May | < 0.0001 | 2017Jul-2018Mar | 0.0001 | 2017Aug-2018Jun | 0.0047 |
| 2018Mar-2017May | < 0.0001 | 2017Oct-2018Mar | 0.0067 | 2018Jan-2018Jun | 0.0005 |
| 2018May-2017May | < 0.0001 | 2018May-2018Mar | 0.0069 | 2017Jun-2018Jun | 0.0001 |
| 2018Nov-2017May | < 0.0001 | 2018Nov-2018Mar | 0.0438 | 2017Sep-2018Jun | 0.0067 |
| 2018Oct-2017May | < 0.0001 | 2018Oct-2018Mar | 0.0180 | 2018May-2018Jun | 0.0496 |
| 2018Sep-2017May | < 0.0001 | 2018Aug-2018Mar | < 0.0001 | 2018Nov-2018Jun | 0.0080 |
| 2019Apr-2017May | < 0.0001 | 2018Feb-2018Mar | 0.0020 | 2018Oct-2018Jun | 0.0208 |
| 2019Feb-2017May | < 0.0001 | 2018Jul-2018Mar | 0.0006 | 2018Sep-2018Jun | < 0.0001 |
| 2019Jan-2017May | < 0.0001 | 2018Jun-2018Mar | < 0.0001 | 2019Apr-2018Jun | 0.0011 |
| 2019Mar-2017May | < 0.0001 | 2018Aug-2018Apr | < 0.0001 | 2019Jan-2018Jun | 0.0007 |
| 2017Jul-2017Nov | 0.0345 | 2018Feb-2018Apr | 0.0161 | 2019Mar-2018Jun | < 0.0001 |
| 2018Aug-2017Nov | 0.0009 | 2017Jul-2018Apr | 0.0011 | | |

**Diversity**

| **Month** | ***p*** | **Month** | ***p*** | **Month** | ***p*** |
|---|---|---|---|---|---|
| 2017Aug-2017Dec | 0.0019 | 2018Oct-2017Dec | < 0.0001 | 2017Sep-2018Mar | 0.0271 |
| 2017Jul-2017Dec | 0.0056 | 2019Apr-2017Dec | < 0.0001 | 2018Nov-2018Mar | 0.0086 |
| 2017Nov-2017Dec | 0.0004 | 2019Feb-2017Dec | 0.0376 | 2019Jan-2018Oct | 0.0454 |
| 2017Oct-2017Dec | 0.0090 | 2019Mar-2017Dec | < 0.0001 | 2019Jan-2019Apr | 0.0204 |
| 2017Sep-2017Dec | < 0.0001 | 2017Sep-2017May | 0.0384 | 2017May-2019Apr | 0.0006 |
| 2018Apr-2017Dec | 0.0008 | 2018Oct-2017May | 0.0016 | 2018Feb-2019Apr | 0.0154 |
| 2018Aug-2017Dec | 0.0135 | 2017Sep-2018Jan | 0.0333 | 2018Oct-2018Mar | 0.0010 |
| 2018Jul-2017Dec | 0.0003 | 2018Nov-2018Jan | 0.0108 | 2019Apr-2018Mar | 0.0003 |
| 2018Jun-2017Dec | 0.0500 | 2018Oct-2018Jan | 0.0013 | 2019Mar-2018Mar | 0.0140 |
| 2018May-2017Dec | < 0.0001 | 2019Mar-2018Jan | 0.01746 | 2019Mar-2018Mar | 0.0140 |
| 2018Nov-2017Dec | < 0.0001 | 2019Apr-2018Jan | 0.0005 | 2018Oct-2018Feb | 0.0351 |

Table S7. Significant results (α-corrected) of pair-wise PERMANOVAs to assess differences in recruit assemblage composition (total number of recruits) between months.

| Assemblage composition | | | | | |
|---|---|---|---|---|---|
| **Month** | ***p*** | **Month** | ***p*** | **Month** | ***p*** |
| 2017Jun-2017Jul | 0.005 | 2017Sep-2019Feb | 0.007 | 2018Mar-2018Aug | 0.010 |
| 2017Jun-2017Aug | 0.006 | 2017Sep-2019Mar | 0.012 | 2018Mar-2018Sep | 0.004 |
| 2017Jun-2017Sep | 0.006 | 2017Sep-2019Apr | 0.004 | 2018Mar-2018Oct | 0.007 |
| 2017Jun-2017Oct | 0.004 | 2017Oct-2017Dec | 0.006 | 2018Mar-2018Nov | 0.006 |
| 2017Jun-2017Nov | 0.009 | 2017Oct-2018Feb | 0.012 | 2018Mar-2019Jan | 0.009 |
| 2017Jun-2017Dec | 0.011 | 2017Oct-2018Apr | 0.007 | 2018Mar-2019Feb | 0.012 |
| 2017Jun-2018Jan | 0.012 | 2017Oct-2018May | 0.009 | 2018Mar-2019Mar | 0.007 |
| 2017Jun-2018Feb | 0.007 | 2017Oct-2018Jun | 0.007 | 2018Mar-2019Apr | 0.009 |
| 2017Jun-2018Mar | 0006 | 2017Oct-2018Aug | 0.005 | 2018Apr-2018Jun | 0.008 |
| 2017Jun-2018Apr | 0.009 | 2017Oct-2018Sep | 0.007 | 2018Apr-2018Jul | 0.005 |
| 2017Jun-2018May | 0.007 | 2017Oct-2018Oct | 0.009 | 2018Apr-2018Aug | 0.007 |
| 2017Jun-2018Jun | 0.005 | 2017Oct-2018Nov | 0.012 | 2018Apr-2018Sep | 0.005 |
| 2017Jun-2018Jul | 0.009 | 2017Oct-2019Jan | 0.005 | 2018Apr-2018Oct | 0.006 |
| 2017Jun-2018Aug | 0.004 | 2017Oct-2019Feb | 0.010 | 2018Apr-2018Nov | 0.002 |
| 2017Jun-2018Sep | 0.013 | 2017Oct-2019Apr | 0.007 | 2018Apr-2019Jan | 0.011 |
| 2017Jun-2018Oct | 0.009 | 2017Nov-2018Jan | 0.011 | 2018Apr-2019Feb | 0.011 |
| 2017Jun-2018Nov | 0.010 | 2017Nov-2018Feb | 0.006 | 2018Apr-2019Mar | 0.009 |
| 2017Jun-2019Jan | 0.010 | 2017Nov-2018Mar | 0.010 | 2018Apr-2019Apr | 0.007 |
| 2017Jun-2019Feb | 0.011 | 2017Nov-2018Apr | 0.009 | 2018May-2018Jun | 0.008 |
| 2017Jun-2019Mar | 0.010 | 2017Nov-2018Jun | 0.006 | 2018May-2018Jul | 0.007 |
| 2017Jun-2019Apr | 0.011 | 2017Nov-2018Jul | 0.010 | 2018May-2018Aug | 0.010 |
| 2017Jul-2017Oct | 0.006 | 2017Nov-2018Aug | 0.008 | 2018May-2018Sep | 0.012 |
| 2017Jul-2017Nov | 0.011 | 2017Nov-2018Sep | 0.005 | 2018May-2018Oct | 0.021 |
| 2017Jul-2017Dec | 0.009 | 2017Nov-2018Oct | 0.012 | 2018May-2019Jan | 0.007 |
| 2017Jul-2018Jan | 0.009 | 2017Nov-2018Nov | 0.011 | 2018May-2019Feb | 0.009 |
| 2017Jul-2018Feb | 0.014 | 2017Nov-2019Jan | 0.007 | 2018May-2019Apr | 0.009 |
| 2017Jul-2018Mar | 0.005 | 2017Nov-2019Feb | 0.008 | 2018Jun-2018Jul | 0.010 |
| 2017Jul-2018Apr | 0.005 | 2017Nov-2019Apr | 0009 | 2018Jun-2018Sep | 0.006 |
| 2017Jul-2018May | 0.008 | 2017Dec-2018Feb | 0.010 | 2018Jun-2018Oct | 0.005 |
| 2017Jul-2018Jun | 0.011 | 2017Dec-2018Mar | 0.016 | 2018Jun-2018Nov | 0.011 |
| 2017Jul-2018Jul | 0.010 | 2017Dec-2018Apr | 0.011 | 2018Jun-2019Jan | 0.011 |
| 2017Jul-2018Aug | 0.012 | 2017Dec-2018May | 0.005 | 2018Jun-2019Feb | 0.007 |
| 2017Jul-2018Sep | 0.012 | 2017Dec-2018Jun | 0.012 | 2018Jun-2019Mar | 0.011 |
| 2017Jul-2018Oct | 0.007 | 2017Dec-2018Jul | 0.012 | 2018Jun-2019Apr | 0.007 |
| 2017Jul-2018Nov | 0.008 | 2017Dec-2018Aug | 0.010 | 2018Jul-2018Aug | 0.008 |
| 2017Jul-2019Mar | 0.006 | 2017Dec-2018Sep | 0.009 | 2018Jul-2018Sep | 0.007 |
| 2017Jul-2019Apr | 0.012 | 2017Dec-2018Oct | 0.012 | 2018Jul-2018Oct | 0.013 |
| 2017Aug-2017Oct | 0.013 | 2017Dec-2018Nov | 0.009 | 2018Jul-2019Jan | 0.012 |
| 2017Aug-2017Nov | 0.010 | 2017Dec-2019Jan | 0.013 | 2018Jul-2019Feb | 0.010 |
| 2017Aug-2017Dev | 0.012 | 2017Dec-2019Feb | 0.006 | 2018Jul-2019Apr | 0.007 |
| 2017Aug-2018Jan | 0.010 | 2017Dec-2019Apr | 0.008 | 2018Aug-2018Sep | 0.008 |
| 2017Aug-2018Feb | 0.010 | 2018Jan-2018Mar | 0.006 | 2018Aug-2018Nov | 0.011 |
| 2017Aug-2018Mar | 0.008 | 2018Jan-2018Apr | 0.004 | 2018Aug-2019Jan | 0.012 |
| 2017Aug-2018Apr | 0.009 | 2018Jan-2018May | 0.006 | 2018Aug-2019Feb | 0.008 |
| 2017Aug-2018Jun | 0.006 | 2018Jan-2018Jun | 0.009 | 2018Aug-2019Mar | 0.005 |
| 2017Aug-2018Jul | 0.007 | 2018Jan-2018Jul | 0.007 | 2018Aug-2019Apr | 0.013 |
| 2017Aug-2018Aug | 0.011 | 2018Jan-2018Aug | 0.012 | 2018Sep-2018Nov | 0.007 |
| 2017Aug-2018Oct | 0.006 | 2018Jan-2018Oct | 0.007 | 2018Sep-2019Jan | 0.012 |

| | | | | | |
|---|---|---|---|---|---|
| 2017Aug-2018Nov | 0.008 | 2018Jan-2018Nov | 0.013 | 2018Sep-2019Feb | 0.011 |
| 2017Aug-2019Jan | 0.012 | 2018Jan-2019Jan | 0.014 | 2018Sep-2019Mar | 0.007 |
| 2017Aug-2019Feb | 0.007 | 2018Jan-2019Mar | 0.002 | 2018Sep-2019Apr | 0.004 |
| 2017Aug-2019Mar | 0.010 | 2018Feb-2018Mar | 0.010 | 2018Oct-2018Nov | 0.004 |
| 2017Aug-2019Apr | 0.010 | 2018Feb-2018Apr | 0.012 | 2018Oct-2019Jan | 0.007 |
| 2017Sep-2017Oct | 0.010 | 2018Feb-2018May | 0.008 | 2018Oct-2019Feb | 0.010 |
| 2017Sep-2017Nov | 0.006 | 2018Feb-2018Jun | 0.008 | 2018Oct-2019Mar | 0.013 |
| 2017Sep-2017Dec | 0.005 | 2018Feb-2018Jul | 0.011 | 2018Oct-2019Apr | 0.006 |
| 2017Sep-2018Jan | 0.009 | 2018Feb-2018Aug | 0.008 | 2018Nov-2019Feb | 0.007 |
| 2017Sep-2018Feb | 0.009 | 2018Feb-2018Nov | 0.009 | 2018Nov-2019Mar | 0.009 |
| 2017Sep-2018Mar | 0.010 | 2018Feb-2019Jan | 0.017 | 2018Nov-2019Apr | 0.006 |
| 2017Sep-2018Apr | 0.009 | 2018Feb-2019Feb | 0.010 | 2019Jan-2019Feb | 0.012 |
| 2017Sep-2018Jun | 0.009 | 2018Feb-2019Mar | 0.006 | 2019Jan-2019Mar | 0.011 |
| 2017Sep-2018Jul | 0.005 | 2018Feb-2019Apr | 0.006 | 2019Jan-2019Apr | 0.009 |
| 2017Sep-2018Aug | 0.007 | 2018Mar-2018Apr | 0.009 | 2019Feb-2019Mar | 0.008 |
| 2017Sep-2018Sep | 0.010 | 2018Mar-2018May | 0.010 | 2019Feb-2019Apr | 0.011 |
| 2017Sep-2018Nov | 0.005 | 2018Mar-2018Jun | 0.004 | 2019Mar-2019Apr | 0.009 |
| 2017Sep-2019Jan | 0.005 | 2018Mar-2018Jul | 0.012 | | |

Table S8. Results of 3-way analyses of variance to compare the differences in recruit abundance per taxa (number of recruits) between months, seasons, and years. Note: F for statistic, significant results ($p < 0.05$) are in bold.

| | | Snails | | | | Limpets | | | |
|---|---|---|---|---|---|---|---|---|---|
| | *df* | SS | MS | F | *p* | SS | MS | F | *p* |
| Year | 1 | 0.31 | 0.31 | 0.16 | 0.6831 | 5.787 | 5.78 | 2.60 | 0.1094 |
| Season | 3 | 21.13 | 7.04 | 3.77 | **0.0128** | 36.63 | 12.21 | 5.49 | **0.0015** |
| Month | 2 | 11.51 | 5.75 | 3.08 | **0.0498** | 29.06 | 14.53 | 6.54 | **0.0020** |
| Year*Season | 3 | 0.86 | 3.28 | 1.76 | 0.1586 | 6.57 | 2.19 | 0.98 | 0.4019 |
| Residuals | 105 | 195.82 | 1.86 | | | 233.19 | 2.22 | | |
| | | **Holothurians** | | | | **Polychaetes** | | | |
| | *df* | SS | MS | F | *p* | SS | MS | F | *p* |
| Year | 1 | 25.53 | 25.53 | 15.17 | **0.0001** | 3.94 | 3.94 | 1.34 | 0.2495 |
| Season | 3 | 39.79 | 13.26 | 7.88 | **< 0.0001** | 214.6 | 71.56 | 24.30 | **< 0.0001** |
| Month | 2 | 0.61 | 0.30 | 0.18 | 0.8349 | 7.21 | 3.60 | 1.22 | 0.2983 |
| Year*Season | 3 | 140.69 | 46.89 | 27.87 | **< 0.0001** | 27.01 | 9.00 | 3.05 | **0.0315** |
| Residuals | 105 | 176.67 | 1.68 | | | 309.20 | 2.94 | | |
| | | **Crabs and shrimps** | | | | **Bivalves** | | | |
| | *df* | SS | MS | F | *p* | SS | MS | F | *p* |
| Month | 1 | 2.48 | 2.48 | 1.10 | 0.2958 | 2.48 | 2.48 | 1.10 | 0.2958 |
| Season | 3 | 22.09 | 7.36 | 2.26 | **0.0242** | 22.09 | 7.36 | 3.26 | **0.0242** |
| Month | 2 | 15.26 | 7.63 | 3.38 | **0.0374** | 15.26 | 7.63 | 3.38 | **0.3749** |
| Year*Season | 3 | 30.61 | 10.20 | 4.52 | **0.0050** | 30.61 | 10.20 | 4.51 | **0.0050** |
| Residuals | 105 | 236.53 | 2.25 | | | 236.53 | 2.25 | | |

Table S9. Significant results of Tukey HSD pair-wise comparisons to assess differences in recruit assemblage abundances per taxa (number of recruits) between months.

**Snail**

| Month | *p* | Month | *p* | Month | *p* |
|---|---|---|---|---|---|
| 2017Jun-2017Jul | < 0.0001 | 2018Jan-2017May | 0.0077 | 2018Jun-2017Nov | 0.0173 |
| 2017Nov-2017Jul | < 0.0001 | 2018Jul-2017May | < 0.0001 | 2018May-2017Nov | 0.0289 |
| 2017Oct-2017Jul | 0.0003 | 2018Jun-2017May | 0.0072 | 2017Sep-2018Apr | 0.0059 |
| 2018Apr-2017Jul | < 0.0001 | 2018Mar-2017May | 0.0007 | 2018Aug-2018Apr | 0.0001 |
| 208Sep-2017Jul | 0.0012 | 2018May-2017May | 0.0041 | 2018Feb-2018Apr | < 0.0001 |
| 2019Mar-2017Jul | 0.0060 | 2018Nov-2017May | 0.0006 | 2018Jan-2018Apr | < 0.0001 |
| 2017May-2017Jun | < 0.0001 | 2018Oct-2017May | < 0.0001 | 2018Jul-2018Apr | 0.0071 |
| 2018Feb-2017Jun | 0.0044 | 2018Sep-2017May | < 0.0001 | 2018Jun-2018Apr | < 0.0001 |
| 2018Jan-2017Jun | 0.03108 | 2019Apr-2017May | < 0.0001 | 2018Mar-2018Apr | 0.0008 |
| 2018Jun-2017Jun | 0.0331 | 2019Feb-2017May | < 0.0001 | 2018May-2018Apr | 0.0001 |
| 2017Nov-2017May | < 0.0001 | 2019Jan-2017May | 0.0001 | 2018Nov-2018Apr | 0.0010 |
| 2017Oct-2017May | < 0.0001 | 2019Mar-2017May | < 0.0001 | 2019Apr-2018Apr | 0.0072 |
| 2017Aug-2017May | 0.0002 | 2017Dec-2017Nov | 0.0009 | 2019Feb-2018Apr | 0.0196 |
| 2017Sep-2017May | < 0.0001 | 2018Aug-2017Nov | 0.0341 | 2017Dec-2018Apr | < 0.0001 |
| 2018Apr-2017May | < 0.0001 | 2018Feb-2017Nov | 0.0021 | 2019Jan-2018Apr | 0.0043 |
| 2018Aug-2017May | 0.0033 | 2018Jan-2017Nov | 0.0162 | | |

**Limpets**

| Month | *p* | Month | *p* | Month | *p* |
|---|---|---|---|---|---|
| 2017Dec-2017Aug | 0.0005 | 2018Oct-2017Jun | 0.0023 | 2018Jul-2018Feb | < 0.0001 |
| 2017Jan-2017Aug | 0.0033 | 2019Jan-2017Jun | 0.0380 | 2018May-2018Feb | 0.0450 |
| 2017May-2017Aug | 0.0005 | 2017Sep-2017May | < 0.0001 | 2018Nov-2018Feb | < 0.0001 |
| 2017Nov-2017Aug | 0.0104 | 2018Jul-2017May | < 0.0001 | 2018Oct-2018Feb | 0.0216 |
| 2018Feb-2017Aug | 0.0289 | 2018May-2017May | 0.0010 | 2018Jul-2018Jan | < 0.0001 |
| 2018Jan-2017Aug | 0.0040 | 2018Nov-2017May | < 0.0001 | 2018May-2018Jan | 0.0067 |
| 2018Jun-2017Aug | 0.0074 | 2018Oct-2017May | 0.0003 | 2018Nov-2018Jan | < 0.0001 |
| 2018Sep-2017Aug | 0.0117 | 2019Jan-2017May | 0.0084 | 2018Oct-2018Jan | 0.0028 |
| 2019Mar-2017Aug | 0.0066 | 2017Sep-2017Nov | < 0.0001 | 2019Jan-2018Jan | 0.0445 |
| 2017Jul-2017Dec | 0.0010 | 2018Jul-2017Nov | < 0.0001 | 2018Jun-2018Jul | < 0.0001 |
| 2017Sep-2017Dec | < 0.0001 | 2018May-2017Nov | 0.0169 | 2018Sep-2018Jul | < 0.0001 |
| 2018Jul-2017Dec | < 0.0001 | 2018Nov-2017Nov | < 0.0001 | 2019Abr-2018Jul | 0.0022 |
| 2018May-2017Dec | 0.0010 | 2018Oct-2017Nov | 0.0076 | 2019Feb-2018Jul | 0.0002 |
| 2018Nov-2017Dec | < 0.0001 | 2018Apr-2017Sep | 0.0114 | 2019Mar-2018Jul | < 0.0001 |
| 2018Oct-2017Dec | 0.0003 | 2018Aug-2017Sep | 0.0003 | 2018May-2018Jun | 0.0122 |
| 2019Jan-2017Dec | 0.0084 | 2018Feb-2017Sep | < 0.0001 | 2018Nov-2018Jun | < 0.0001 |
| 2017Jul-2017Jul | 0.0059 | 2018Jan-2017Sep | < 0.0001 | 2018Sep-2018Jun | 0.0053 |
| 2017May-2017Jul | 0.0010 | 2018Jun-2017Sep | < 0.0001 | 2018Sep-2018May | 0.0190 |
| 2017Nov-2017Jul | 0.0178 | 2018Sep-2017Sep | < 0.0001 | 2018Mar-2018May | 0.0110 |
| 2018Feb-2017Jul | 0.0471 | 2019Apr-2017Sep | 0.0012 | 2018Sep-2018Nov | < 0.0001 |
| 2018Jan-2017Jul | 0.0071 | 2019Feb-2017Sep | 0.0001 | 2019Abr-2018Nov | 0.0022 |
| 2018Jun-2017Jul | 0.0128 | 2019Mar-2017Sep | < 0.0001 | 2019Feb-2018Nov | 0.0002 |
| 2018Sep-2017Jul | 0.0199 | 2018Jul-2018Apr | 0.0189 | 2019Mar-2018Nov | < 0.0001 |
| 2018Jun-2017Jun | < 0.0001 | 2018Nov-2018Apr | 0.0185 | 2018Sep-2018Oct | 0.0086 |
| 2018May-2017Jun | 0.0056 | 2018Jul-2018Aug | 0.0007 | 2019Feb-2018Oct | 0.0456 |
| 2018Nov-2017Jun | < 0.0001 | 2018Nov-2018Aug | 0.0006 | 2019Mr-2018Oct | 0.0048 |

| **Crabs and Shrimps** | **Bivalves** |
|---|---|

| Month | *p* | Month | *p* | Month | *p* |
|---|---|---|---|---|---|
| 2018Mar-2017May | 0.0158 | 2018Sep-2017Aug | 0.0082 | | |
| 2018Mar-2017Nov | 0.0039 | 2019Apr-2017Aug | 0.03947 | | |
| 2018Feb-2017Oct | 0.0212 | 2018Sep-2017Dec | 0.04741 | | |
| 2018Mar-2017Oct | 0.0021 | 2018Sep-2017Oct | 0.0083 | | |
| 2018Mar-2018Jan | 0.0165 | 2019Apr-2017Oct | 0.0397 | | |
| 2018Mar-2018Jul | 0.0439 | 2018Sep-2018Feb | 0.0003 | | |
| 2018Mar-2018Jun | 0.0079 | 2019Apr-2018Feb | 0.0022 | | |
| 2019Jan-2018Mar | 0.0065 | 2018Sep-2018Jan | 0.0431 | | |
| | | 2018Sep-2018Jun | 0.0020 | | |
| | | 2019Apr-2018Jun | 0.0114 | | |
| | | 2018Sep-2018Mar | 0.0068 | | |
| | | 2019Apr-2018Mar | 0.0336 | | |
| | | 2018Sep-2018May | 0.0177 | | |
| | | 2019Feb-2018Sep | 0.0136 | | |
| | | 2019Jan-2018Sep | 0.0028 | | |
| | | 2019Jan-2019Apr | 0.0154 | | |

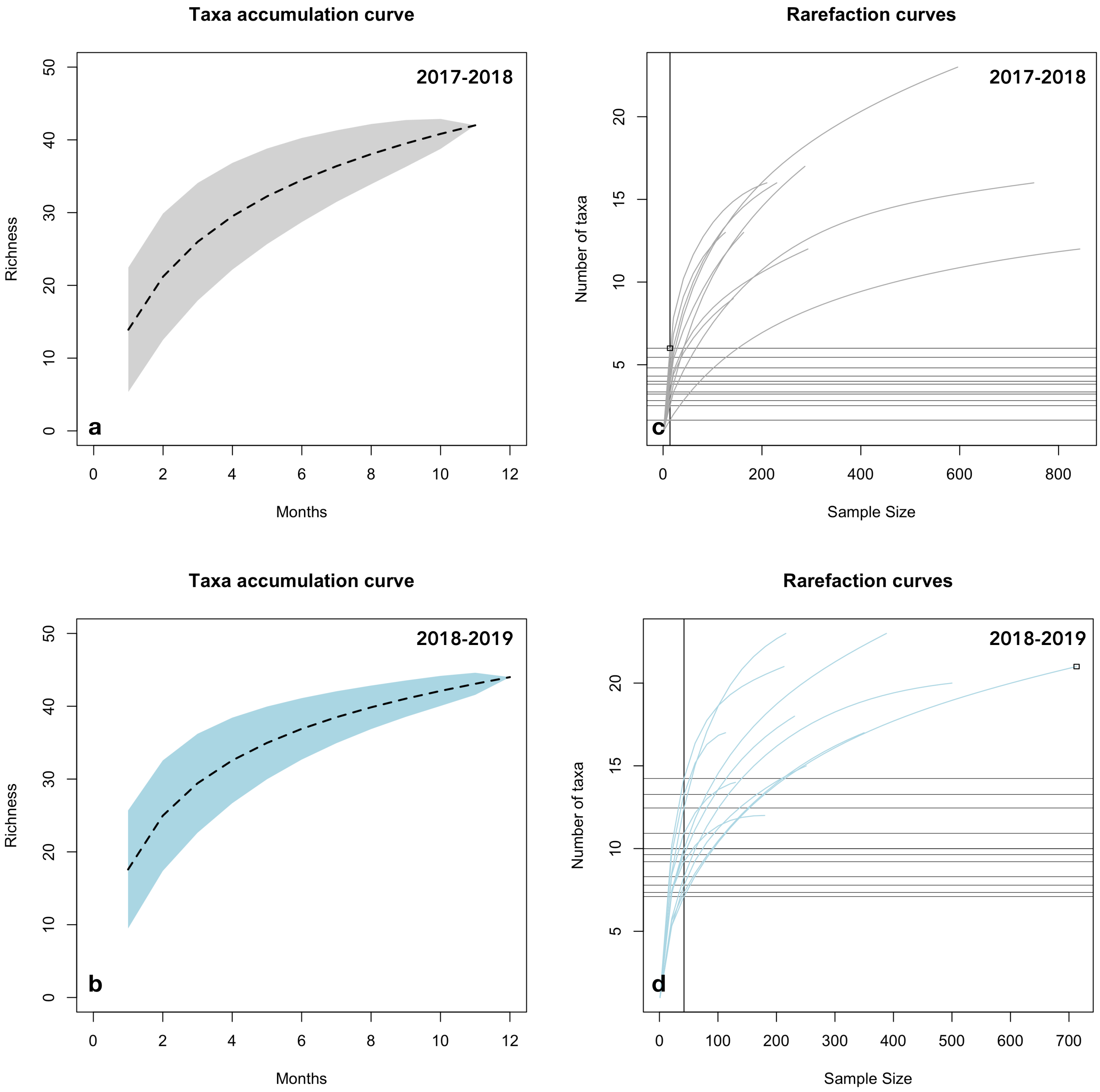


Figure S4. Comparison of sampling representativeness (taxa accumulation, a, b; rarefaction curve, c, d) in the sampling years (2017-2018, 2018-2019). Rarefaction curves represent monthly samplings.

Table S10. Results of the cross correlation analysis comparing monthly variability in recruit abundance assemblage abundance (total number of recruits), composition (number of recruits per taxa), diversity (Shannon-Wiener Index S-W), and richness (number of species/taxa) with the Seascape patterns (Coverage, relative amount of pixels classified in each Seascape). Significant time-lagged correlations were also included. Terms: r is the correlation coefficient; lag is the time lag (months); nc not correlated. Significant results * ($p \leq 0.01$ and lag 0 to -2 months) are in bold.

| Seascape category | Abundance | | Diversity | | Richness | |
|---|---|---|---|---|---|---|
| | r | lag | r | lag | r | lag |
| **3** | 0.69 | -5 | -0.53 | -5 | nc | nc |
| **5** | **0.51** | **-1** | nc | nc | nc | nc |
| **13** | 0.49 | -7 | 0.44 | 4 | 0.41 | -3 |
| **15** | **0.49** | **+1** | nc | nc | -0.47 | +4 |
| | -0.62 | +3 | nc | nc | 0.43 | -4 |
| **21** | 0.43 | +3 | nc | nc | **-0.45** | **0** |
| | 0.52 | -6 | nc | nc | nc | nc |
| | 0.41 | -7 | nc | nc | nc | nc |
| **27** | 0.43 | -9 | nc | nc | nc | nc |

| | Snails | | Limpets | | Holothurians | | Polychaetes | | Crabs and shrimps | | Bivalves | |
|---|---|---|---|---|---|---|---|---|---|---|---|---|
| | r | lag | r | lag | r | lag | r | lag | r | lag | r | lag |
| 3 | **0.64** | **-1** | | | 0.42 | -9 | 0.75 | -5 | | | 0.66 | +4 |
| 5 | **0.64** | **-1** | 0.48 | -8 | | | | | | | 0.54 | +4 |
| | | | | | | | | | | | 0.56 | -5 |
| | | | | | | | | | | | 0.45 | -6 |
| 13 | 0.54 | -3 | | | | | 0.49 | -7 | | | **0.62** | **+2** |
| 15 | **0.41** | **+1** | 0.44 | +8 | | | -0.51 | +3 | | | 0.46 | +6 |
| | 0.42 | -4 | | | | | 0.45 | -3 | | | | |
| 21 | | | | | | | 0.46 | +3 | | | -0.48 | -4 |
| 27 | 0.42 | +3 | | | | | 0.49 | -6 | **0.44** | **+1** | 0.52 | -3 |
| | | | | | | | | | | | 0.59 | -4 |

* Tropical-Subtropical Transition (3), Subtropical Gyre Transition (5), Subtropical Gyre Mesoscale Influenced (13), Tropical Seas (15), Warm, Blooms, High Nuts (21), and Hypersaline Eutrophic (27).

Table S11. Results of canonical analyses of principal coordinates (CAP) to evaluate the contribution of the local Seascape patterns (Coverage, relative amount of pixels classified in each Seascape) to month variations in the benthic assemblage composition (abundance per taxa) at the study site. Spearman correlation values for each environmental variable are described for in CAP axis 1-2. Note: proportion of variability explained by CAP axes are between parentesis '()', F for statistic, significant results ($p < 0.05$) are in bold.

| | F = 1.11, *p* = 0.267 | | | |
|---|---|---|---|---|
| Seascape Category | CAP 1 (51%) | CAP 2 (23%) | F | *p* |
| 3 Tropical-Subtropical Transition | -0.22 | 0.49 | 0.82 | 0.612 |
| 5 Subtropical Gyre Transition | -0.09 | 0.64 | 0.92 | 0.513 |
| 13 Subtropical Gyre Mesoscale Influenced | 0.23 | -0.63 | 1.03 | 0.392 |
| 15 Tropical Seas | 0.27 | 0.42 | 0.69 | 0.762 |
| 21 Warm, Blooms, High Nuts | -0.73 | -0.32 | 2.64 | **0.004** |
| 27 Hypersaline Eutrophic | 0.73 | 0.14 | 0.57 | 0.878 |

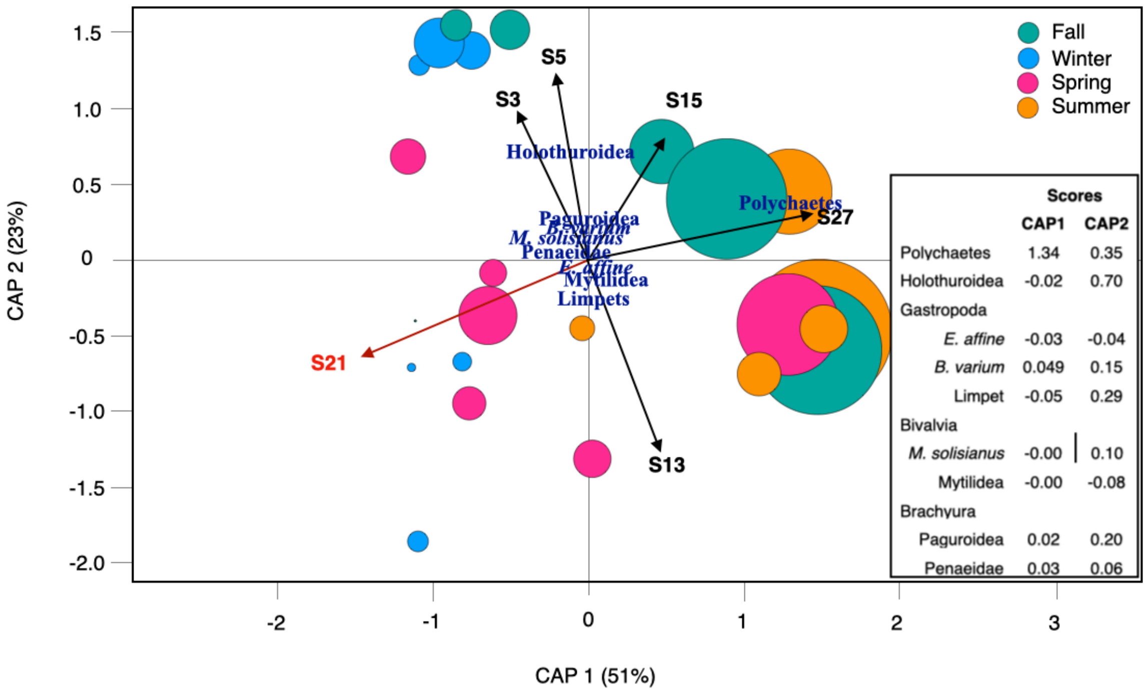


Figure S5. Canonical analyses of principal coordinates (CAP) ordination of samples according to differences in month recruit abundance per taxa (number of recruits per kg of algae) and the contribution of local Seascapes (variability in regional % Cover per class). Vectors are based on Spearman correlation values > 0.5 (p > 0.5) for Seascapes and scores for taxa. Proportion of data explained by axis 1 and 2 are in parenthesis. Note: size of circles represent average abundance of recruits per month, ranked by maximum (850) and minimum (12) values.
